# Ontology and diversity of transcript-associated microsatellites mined from a globe artichoke EST database

**DOI:** 10.1186/1471-2164-10-454

**Published:** 2009-09-28

**Authors:** Davide Scaglione, Alberto Acquadro, Ezio Portis, Christopher A Taylor, Sergio Lanteri, Steven J Knapp

**Affiliations:** 1 Di.Va.P.R.A. Plant Genetics and Breeding, University of Torino, via L. da Vinci 44, 10095 Grugliasco (Torino), Italy; 2 Institute for Plant Breeding, Genetics, and Genomics, University of Georgia, 111 Riverbend Rd., 30602 Athens, Georgia, USA

## Abstract

**Background:**

The globe artichoke (*Cynara cardunculus *var. *scolymus *L.) is a significant crop in the Mediterranean basin. Despite its commercial importance and its both dietary and pharmaceutical value, knowledge of its genetics and genomics remains scant. Microsatellite markers have become a key tool in genetic and genomic analysis, and we have exploited recently acquired EST (expressed sequence tag) sequence data (Composite Genome Project - CGP) to develop an extensive set of microsatellite markers.

**Results:**

A unigene assembly was created from over 36,000 globe artichoke EST sequences, containing 6,621 contigs and 12,434 singletons. Over 12,000 of these unigenes were functionally assigned on the basis of homology with *Arabidopsis thaliana *reference proteins. A total of 4,219 perfect repeats, located within 3,308 unigenes was identified and the gene ontology (GO) analysis highlighted some GO term's enrichments among different classes of microsatellites with respect to their position. Sufficient flanking sequence was available to enable the design of primers to amplify 2,311 of these microsatellites, and a set of 300 was tested against a DNA panel derived from 28 *C. cardunculus *genotypes. Consistent amplification and polymorphism was obtained from 236 of these assays. Their polymorphic information content (PIC) ranged from 0.04 to 0.90 (mean 0.66). Between 176 and 198 of the assays were informative in at least one of the three available mapping populations.

**Conclusion:**

EST-based microsatellites have provided a large set of *de novo *genetic markers, which show significant amounts of polymorphism both between and within the three taxa of *C. cardunculus*. They are thus well suited as assays for phylogenetic analysis, the construction of genetic maps, marker-assisted breeding, transcript mapping and other genomic applications in the species.

## Background

The globe artichoke *Cynara cardunculus *is a member of the Asteraceae (Compositae) family, and originates from the Mediterranean basin [1]. The species is subdivided into three taxa - the globe artichoke (var. *scolymus *L.), the cultivated cardoon (var. *altilis *DC), and their progenitor, the wild cardoon [var. *sylvestris *(Lamk) Fiori]. The edible part of the globe artichoke plant is provided by its immature inflorescence, referred as a capitulum or head [2], and represents a significant component of the Mediterranean diet. The cultivated cardoon is grown for its fleshy stems, which are used in traditional cuisine. Leaf extracts of the species contain a number of bioactive compounds (e.g., quercetin, rutin, luteolin, gallic acid, di-caffeoylchinic acid, and sesquiterpene lactones) which have been shown to have anti-oxidative and anti-carcinogenic activity, to inhibit cholesterol biosynthesis, and to enhance lipid metabolism [3-8]. The antioxidant content per serving of globe artichoke ranks very highly among vegetables [9], while the roots provide a source of inulin, a proven enhancer of human intestinal flora [10,11]. In spite of its economic importance, however, little breeding effort has been applied to date in the globe artichoke.

Progress has been made in the development of DNA marker based genetic maps in globe artichoke [12-14]. The most saturated map has been recently developed from a set of F_1 _progeny of a cross between a globe artichoke and a cultivated cardoon genotypes [14]. This map consisted of 20 linkage groups comprising 326 loci and spanned ~1500 cM with a mean inter-marker distance of ~4.5 cM. A set of loci common to this map and a previously developed one [12] allowed for map alignment and the definition of 17 homologous linkage groups, corresponding to the haploid chromosome number of the species.

It was long assumed that SSRs were primarily associated with non-coding DNA, but it has now become clear that they are more frequent in transcribed than non-transcribed sequences and equally frequent in the transcriptomes of plants with dramatically different nuclear DNA contents [15]. EST databases therefore represent a convenient resource for the identification of microsatellites, some of which, as a result of their presence within coding DNA, have the potential to deliver informative within gene markers, exploitable as COS (conserved orthologous set) for genomic comparative analysis.

Here, we report: i) the unigene assembly based on the globe artichoke EST database deposited in GenBank by the Compositae Genome Project (CGP), ii) the identification of a wide set of EST-based microsatellite markers and iii) the evaluation of the informativeness of a subset of these markers using a panel of *C. cardunculus *genotypes. Furthermore, we performed a comprehensive functional annotation, inferred from sequence alignment (ISA), as well as a gene ontology categorisation inferred from sequence orthology (ISO) of the SSR-containing unigenes. At last we assessed whether motif type and relative position within CDSs (*Coding DNA Sequences*)/UTRs (*Untraslated Regions*) may be preferentially associated with a particular gene ontology term.

## Results and Discussion

### EST microsatellite discovery, frequency and primer design

**Figure 1:**
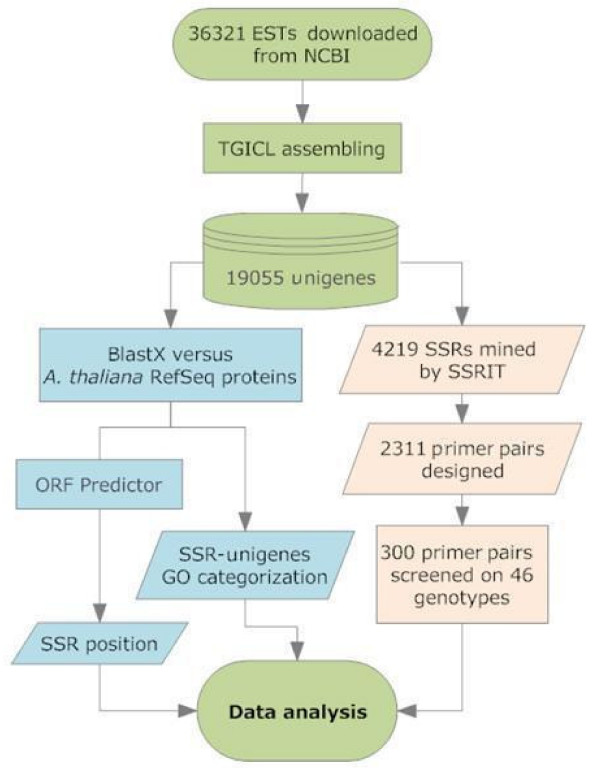
**The schema used for EST assembly, annotation, primer design and amplicon screening**.

**Figure 2:**
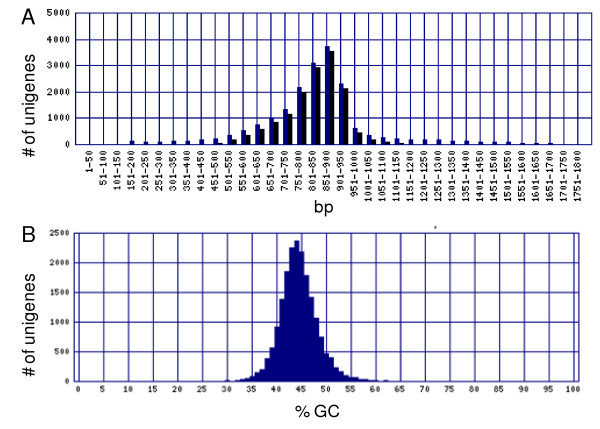
**Output of the EST assembly**. Distribution of unigene (A) length, and (B) GC-content.

Globe artichoke ESTs were trimmed, assembled, and annotated using a customized bioinformatic pipeline (Figure 1) into 19,055 unigenes (6,621 contigs and 12,434 singletons) spanning 15 Mbp. The transcript assembly and unigene consensus sequences are supplied as electronic supplementary materials (See Additional file 1, 2, 3: EST assembly, 19,055 unigenes, ACE assembly file). The unigenes had a mean length of 786 ± 1.7 bp, with a mean GC content of 43.9 ± 0.03% (range 24.7 - 67.9%, Figure 2) and a mean ambiguity code ratio of 0.51 ± 0.01. Within the unigene set, 3,308 contained 4,219 uninterrupted tracts of (perfect) di-, tri-, tetra-, penta-, and hexa-nucleotide repeats, delivering a mean microsatellite density of one per 3.6 kb. Comparable microsatellite frequencies and densities have been discovered in the transcriptomes of other Compositae species [16-18]. Only perfect repeats were selected, as these appear to be the more prone to strand slippage and, consequently, tend to be more polymorphic than interrupted ones [19]. Sufficient flanking sequence (in terms of both length and read quality) was present in 1,974 of the unigenes, containing 2,311 perfect microsatellites. The resulting PCR primers designed for these loci are given in Additional File 4 (primer pairs designed).

### Allelic diversity with the EST microsatellites

A subset of 300 microsatellites (ranging in length from 10 to 84 bp, and representative of a broad spectrum of the repeat types) was surveyed for their informativeness. The targeted amplicon length ranged from 98 to 456 bp and the set was selected to optimize the possibility of multiplexing on the capillary genotyping platform employed. The test germplasm panel consisted of twelve genotypes of globe artichoke, nine of cultivated cardoon, and seven of wild cardoon (Table 1). In all, 238 (79.3%) of the assays were successful; of these, 236 were informative among the taxa, while 215, 216 and 223 were polymorphic within, respectively, globe artichoke, cultivated cardoon and wild cardoon (Table 2 and Additional file 5: full statistics on 300 SSR-containing loci). A total of 1,546 alleles was generated from the 238 successful assays, giving a mean of 3.8 (range 1-15) alleles per locus. The largest range in amplicon length observed was 196 - 252 bp, observed for a TCA_n _microsatellite (CyEM-171). In 85% of the loci, the assay generated the predicted length of amplicon, in 12.2% the amplicon was longer than expected, and in 2.8% it was shorter. The allelic range (in terms of amplicon length) was greater for the wild cardoon (17.1 ± 1.0 bp) than for globe artichoke (13.6 ± 0.8 bp) or cultivated cardoon (13.7 ± 0.7 bp).

**Table 1:** Genotypes set.

***C. cardunculus *taxa**	**Genotypes**	**Cluster**^1^
*scolymus*	Violet de Provence	**A1**
	AVM 7	**A1**
	Blanco	**A1**
	Pasquaiolo	**A2**
	Pietralcina	**A2**
	Romanesco C3	**A2**
	Green Globe	**A2**
	Sakiz	**B1**
	Spinoso di Palermo	**B1**
	Spinoso violetto di Liguria	**B2**
	Empolese	**B2**
	Violetto di Toscana	**B2**
		
*altilis*	Blanco de Peralta	**A1**
	Lleno de España	**A1**
	Rojo de Corella	**A2**
	Valencia	**A3**
	Gigante di Romagna	**B1**
	Bianco Avorio	**B2**
	Gobbo di Nizza	**B2**
	Bianco Pieno Migliorato	**C**
	Altilis 41	**-**
		
*sylvestris*	Bronte	**Sicily**
	Roccella	**Sicily**
	Palazzolo	**Sicily**
	Sassari	**Sardinia**
	Oristano	**Sardinia**
	Nuoro	**Sardinia**
	Creta 4	**-**

The 28 *C. cardunculus *accessions assayed for genotypic variation. Respectively 12 globe artichokes, 9 cultivated cardoons and 7 wild cardoons were analysed.^1^Globe artichoke, cultivated cardoon and wild cardoon clusters defined in [20,21,26], respectively.

**Table 2:** CyEM loci statistics.

**Locus**	**Accession N°/unigene name**	**Exp. Size**	**Motif**	**N° of repeats**	**Allele size range**	**n_a_**	**n_e_**	**H_o_**	**PIC**	**GxG**	**GxC**	**GxW**
**CyEM-001**	GE602982	148	ATAC	5	141-150	3	2,018	0,679	0,504	+	+	+
**CyEM-002**	GE604144	150	TTTG	6	139-143	2	1,733	0,464	0,423	+	+	+
**CyEM-003**	GE604267	144	CAATGG	9	110-141	5	3,324	0,440	0,699	-	+	-
**CyEM-004**	GE604528	175	ATC	20	125-138	3	1,075	0,071	0,070	-	-	-
**CyEM-005**	GE605519	154	TC	9	137-170	9	2,446	0,571	0,591	+	-	+
**CyEM-006**	GE606838	152	ATC	13	135-162	8	3,269	0,556	0,694	-	-	+
**CyEM-007**	GE609770	165	TTC	17	121-165	8	3,735	0,577	0,732	+	+	+
**CyEM-008**	GE610358	149	CAT	19	106-156	9	3,347	0,654	0,701	+	+	+
**CyEM-009**	GE610418	143	ATAC	6	118-146	10	6,693	0,500	0,851	+	+	+
**CyEM-010**	GE612656	180	TGTA	7	152-194	12	7,259	0,643	0,862	+	+	+
**CyEM-011**	GE613193	209	TTGGTT	10	170-214	5	3,057	0,393	0,673	-	+	+
**CyEM-012**	GE578689	153	GAA	8	146-169	8	4,036	0,538	0,752	+	+	+
**CyEM-013**	GE588213	149	GAT	10	124-151	8	3,416	0,429	0,707	+	+	+
**CyEM-014**	GE595596	170	ATG	11	153-172	7	4,308	0,643	0,768	+	+	+
**CyEM-015**	CL2994Contig1	92	GTTT	5	84-97	4	2,592	0,750	0,614	+	+	+
**CyEM-016**	GE587666	127	TATG	5	117-129	4	2,922	0,481	0,658	+	+	+
**CyEM-017**	CL469Contig1	187	TTGGT	6	173-205	7	3,588	0,429	0,721	-	-	+
**CyEM-018**	CL6299Contig1	156	GGTCT	6	139-156	2	1,813	0,321	0,448	-	-	-
CyEM-019*	CL2425Contig1	179	TGGTA	6	-	\	\	\	\	+	+	+
**CyEM-020**	GE612053	107	TCATCT	6	67-104	7	3,724	0,321	0,732	+	+	+
**CyEM-021**	CL8Contig1	231	CGC	5	234-234	1	1,000	0,000	0,000	-	-	-
**CyEM-022**	CL167Contig1	222	ATG	5	224-224	1	1,000	0,000	0,000	-	-	-
**CyEM-023**	CL290Contig1	234	CT	5	214-233	4	1,675	0,429	0,403	-	+	-
**CyEM-024**	CL432Contig1	125	ATC	7	113-126	4	3,655	0,464	0,726	+	+	+
**CyEM-025**	CL489Contig1	229	CTA	6	229-239	3	1,878	0,321	0,467	+	+	+
**CyEM-027**	CL768Contig1	228	ACC	6	224-227	2	1,036	0,036	0,035	-	-	-
**CyEM-028**	CL1480Contig1	228	CGATTA	7	530-544	4	1,845	0,095	0,458	+	-	+
**CyEM-029**	CL2522Contig1	219	CTTC	7	202-223	5	3,315	0,607	0,698	+	+	+
**CyEM-030**	CL2739Contig1	240	TC	19	212-245	11	7,245	0,667	0,862	-	+	+
**CyEM-031**	CL2833Contig1	223	CT	8	117-226	7	3,682	0,333	0,728	+	-	+
**CyEM-032**	CL5674Contig1	229	CAT	8	214-236	5	3,294	0,500	0,696	+	+	+
**CyEM-033**	CL6305Contig1	227	CTT	12	205-231	8	3,213	0,571	0,689	+	-	+
**CyEM-034**	CL6392Contig1	212	CT	17	185-224	9	6,078	0,750	0,835	+	+	+
**CyEM-035**	CL136Contig1	231	GGTTA	5	212-239	6	3,435	0,739	0,709	+	+	+
**CyEM-036**	CL840Contig1	231	GAATT	5	222-237	4	2,379	0,500	0,580	+	+	+
**CyEM-037**	CL1651Contig1	220	CT	16	191-225	12	6,759	0,679	0,852	+	+	+
**CyEM-038**	CL3137Contig1	220	AAGTG	5	216-226	3	2,316	0,536	0,568	+	+	+
**CyEM-042**	CL4773Contig1	168	AG	14	148-176	12	6,438	0,577	0,845	+	+	+
**CyEM-043**	CL5064Contig1	292	ACA	10	272-297	7	4,021	0,500	0,751	+	+	+
**CyEM-045**	CL5134Contig1	301	CAATC	5	290-299	3	1,651	0,280	0,394	+	+	+
**CyEM-046**	CL5445Contig1	278	CTTTGC	5	273-278	2	1,415	0,071	0,293	-	-	+
**CyEM-047**	CL6123Contig1	286	CATCTT	5	274-303	5	3,226	0,571	0,690	+	+	+
**CyEM-048**	CL703Contig1	292	CAATCC	5	270-294	6	3,378	0,560	0,704	+	+	+
**CyEM-049**	CL1527Contig1	305	CAGAAG	6	284-307	5	3,246	0,393	0,692	+	-	-
**CyEM-050**	CL1584Contig1	311	TTGGT	5	269-315	8	1,617	0,240	0,382	-	-	+
**CyEM-051**	CL1735Contig1	162	TGGCAA	5	129-167	5	1,551	0,074	0,355	-	-	-
**CyEM-052**	CL1878Contig1	301	CT	15	277-303	11	6,788	0,964	0,853	+	+	+
**CyEM-053**	CL2037Contig1	327	CT	20	297-330	13	8,667	0,885	0,885	+	+	+
**CyEM-054**	CL4038Contig1	307	ATGTGG	6	268-305	11	9,191	0,600	0,891	+	+	+
**CyEM-055**	CL4185Contig1	297	CAACAG	7	271-293	5	3,995	0,630	0,750	+	+	+
**CyEM-056**	CL5289Contig1	301	GA	21	283-319	9	4,404	0,385	0,773	+	+	+
**CyEM-057**	CL6231Contig1	301	AT	6	300-323	6	2,620	0,308	0,618	+	+	+
**CyEM-058**	CL815Contig1	299	CA	7	291-298	2	1,642	0,000	0,391	-	+	+
**CyEM-059**	CL1157Contig1	289	GGT	9	280-297	7	2,116	0,429	0,527	+	+	+
**CyEM-060**	CL1449Contig1	306	GATTC	5	294-307	4	2,605	0,429	0,616	+	+	+
**CyEM-063**	GE590526	375	GATGG	5	432-457	8	3,673	0,481	0,728	+	+	+
**CyEM-064**	GE590983	122	CT	15	96-126	12	8,145	0,741	0,877	+	+	+
**CyEM-066**	GE591829	368	GAT	15	372-405	10	6,231	0,704	0,840	+	+	+
**CyEM-069**	GE594774	386	AGGA	5	370-392	6	3,556	0,519	0,719	-	+	+
**CyEM-070**	GE594818	272	TGCA	5	309-380	5	3,769	0,393	0,735	+	+	+
**CyEM-071**	GE595888	376	GTTTG	5	438-468	6	2,713	0,357	0,631	-	-	-
**CyEM-072**	GE595959	376	AAGCA	5	367-386	4	3,308	0,536	0,698	+	+	+
**CyEM-073**	GE596794	376	AGCC	6	457-465	4	2,088	0,286	0,521	+	+	-
**CyEM-075**	GE597515	390	TC	16	363-393	7	3,458	0,462	0,711	-	+	+
**CyEM-076**	GE597588	378	AACCA	14	436-449	6	3,627	0,556	0,724	+	+	+
**CyEM-077**	GE598177	378	CCAT	6	370-380	5	3,492	0,429	0,714	+	+	+
**CyEM-079**	GE601502	375	AATG	6	463-488	8	5,333	0,625	0,813	+	+	+
**CyEM-080**	GE602408	382	TTCACG	14	652-694	4	3,273	0,714	0,695	+	+	+
**CyEM-083**	CL5605Contig1	465	AG	13	448-475	7	3,689	0,643	0,729	+	-	-
**CyEM-084**	CL5717Contig1	128	AATCA	5	108-123	3	2,018	0,429	0,504	+	+	+
**CyEM-086**	GE577139	430	ATGTAA	6	410-460	6	3,447	0,389	0,710	+	+	+
**CyEM-087**	GE578205	450	CCAAC	5	443-457	5	1,488	0,125	0,328	-	-	-
**CyEM-088**	GE578232	451	GAGGAA	7	436-459	5	2,045	0,222	0,511	-	-	+
**CyEM-090**	GE580735	201	ATAC	6	190-218	5	1,615	0,222	0,381	-	+	-
**CyEM-091**	GE581152	452	GGTAT	5	657-669	4	2,493	0,464	0,599	+	+	+
**CyEM-092**	GE581504	106	TTGC	7	83-104	6	4,683	0,750	0,786	-	-	-
**CyEM-093**	GE581834	435	GA	18	410-453	12	7,362	0,857	0,864	+	+	+
**CyEM-094**	GE581842	450	TCA	14	417-454	5	2,068	0,333	0,516	+	+	+
**CyEM-096**	GE586326	451	CTCTAT	6	426-465	9	3,144	0,346	0,682	+	-	-
**CyEM-097**	GE587846	446	GT	12	437-449	5	3,415	0,556	0,707	+	+	+
**CyEM-098**	GE588210	262	AAGAG	5	620-650	4	3,068	0,357	0,674	-	-	-
**CyEM-099**	GE588482	448	AAGTG	5	536-547	3	2,594	0,593	0,615	+	+	+
**CyEM-100**	GE589916	434	AT	11	522-554	10	6,857	0,375	0,854	-	-	+
**CyEM-102**	GE590134	100	ACC	7	87-105	5	1,871	0,250	0,466	-	+	+
**CyEM-103**	GE592369	100	AGC	7	93-105	4	1,821	0,571	0,451	+	-	+
**CyEM-104**	GE595980	100	CAG	7	94-117	8	4,226	0,679	0,763	+	+	+
**CyEM-105**	GE588534	101	AAG	7	85-107	6	3,355	0,654	0,702	+	+	+
**CyEM-106**	GE588636	101	CAG	7	84-99	6	3,908	0,692	0,744	+	+	+
**CyEM-107**	GE591921	101	GAA	7	91-115	6	3,391	0,704	0,705	+	+	+
**CyEM-108**	GE590638	102	ACA	7	293-356	12	5,481	0,778	0,818	+	+	+
**CyEM-109**	GE586147	103	GGA	7	50-102	7	3,980	0,357	0,749	-	-	+
**CyEM-110**	GE586350	103	GTT	7	98-105	3	1,618	0,393	0,382	-	+	-
**CyEM-111**	GE587414	165	CGG	7	105-124	6	4,467	0,464	0,776	+	+	+
**CyEM-112**	GE593991	104	TCA	7	99-117	7	4,519	0,679	0,779	+	+	+
**CyEM-113**	GE584535	107	CAC	7	97-102	3	1,332	0,286	0,249	+	+	+
**CyEM-115**	GE582326	109	CTG	7	94-120	6	2,190	0,179	0,543	-	-	-
**CyEM-117**	GE596127	110	GAT	7	103-109	3	1,742	0,429	0,426	+	+	+
**CyEM-118**	GE597580	111	GCT	7	96-117	6	3,197	0,593	0,687	+	+	+
**CyEM-120**	GE597566	113	ATT	7	99-120	6	3,350	0,464	0,702	+	+	+
**CyEM-121**	GE590328	114	ACA	7	265-287	7	3,574	0,519	0,720	+	+	+
**CyEM-122**	GE583054	115	CTG	7	118-133	5	2,149	0,385	0,535	-	+	+
**CyEM-123**	GE592105	115	GTG	7	107-120	3	1,640	0,500	0,390	+	+	+
**CyEM-124**	GE597437	117	GT	12	112-126	8	4,598	0,571	0,783	+	+	+
**CyEM-126**	GE601086	119	AGC	8	111-158	7	2,834	0,731	0,647	-	+	+
**CyEM-127**	GE586328	110	CCA	8	99-116	7	3,960	0,857	0,747	+	+	+
**CyEM-128**	CL4629Contig1	120	AG	12	103-131	10	2,830	0,500	0,647	+	+	+
**CyEM-129**	GE594087	123	AGT	8	105-124	5	2,649	0,250	0,622	-	-	-
**CyEM-130**	GE610344	123	GAT	8	112-131	6	3,168	0,393	0,684	+	+	+
**CyEM-131**	GE580155	258	TC	12	260-296	9	4,989	0,538	0,800	-	-	-
**CyEM-132**	GE589900	126	GTG	8	112-127	5	2,851	0,286	0,649	-	-	+
**CyEM-133**	GE582083	128	CAT	8	121-155	10	3,806	0,464	0,737	-	+	+
**CyEM-134**	GE587520	128	TGA	8	123-129	3	2,402	0,407	0,584	+	+	+
**CyEM-135**	GE580164	129	TC	12	125-151	10	6,426	0,893	0,844	+	+	+
**CyEM-136**	GE599224	129	GA	12	118-145	10	5,502	0,679	0,818	+	+	+
**CyEM-138**	CL2919Contig1	130	TC	12	112-153	13	6,788	0,714	0,853	+	+	+
**CyEM-139**	CL5080Contig1	130	CAA	8	122-139	6	2,835	0,500	0,647	+	-	+
**CyEM-141**	GE588755	133	ATC	8	119-134	5	3,019	0,593	0,669	+	+	+
**CyEM-142**	GE581587	134	CAT	8	119-140	5	2,246	0,231	0,555	-	-	-
**CyEM-143**	GE577330	135	AG	12	125-145	8	3,540	0,357	0,717	-	-	+
**CyEM-144**	GE602230	136	TGA	8	128-158	6	3,853	0,679	0,740	+	+	+
**CyEM-145**	GE594958	139	GAT	8	133-181	8	3,807	0,519	0,737	+	+	+
**CyEM-146**	CL4926Contig1	164	CCA	6	226-241	7	2,259	0,143	0,557	+	+	-
**CyEM-147**	CL2920Contig1	165	CTC	7	140-164	7	2,864	0,286	0,651	+	+	+
**CyEM-148**	GE608083	167	GAT	6	161-179	5	2,420	0,250	0,587	-	+	+
**CyEM-149**	GE605451	168	CA	10	115-124	3	2,378	0,222	0,580	-	-	-
**CyEM-150**	GE588087	169	AG	10	138-171	9	4,681	0,286	0,786	+	+	+
**CyEM-151**	CL1781Contig1	169	CAC	7	150-169	6	3,136	0,429	0,681	+	-	+
**CyEM-152**	GE579243	170	GA	10	165-187	8	4,976	0,407	0,799	+	+	+
**CyEM-153**	GE603802	171	AAC	6	165-183	6	2,450	0,393	0,592	-	+	+
**CyEM-154**	GE581295	102	TC	10	94-106	7	1,653	0,423	0,395	+	-	+
**CyEM-155**	GE608129	173	CAG	6	165-174	4	2,877	0,571	0,652	+	+	+
**CyEM-156**	GE596512	174	TC	10	171-185	6	4,094	0,464	0,756	+	+	+
**CyEM-157**	CL5016Contig1	174	TGA	6	154-197	8	6,698	0,458	0,851	+	+	+
**CyEM-158**	GE599088	200	TCA	6	193-199	2	1,899	0,308	0,473	+	+	+
**CyEM-159**	GE612769	176	TC	10	167-187	9	3,260	0,571	0,693	+	-	+
**CyEM-160**	CL3274Contig1	176	CTT	7	163-185	6	1,589	0,286	0,371	-	-	-
**CyEM-162**	CL1575Contig1	258	TAG	7	253-261	5	2,914	0,500	0,657	+	+	+
**CyEM-163**	GE595958	179	AC	10	174-190	8	4,442	0,607	0,775	+	+	+
**CyEM-164**	GE583509	182	AC	10	380-401	5	3,406	0,440	0,706	+	+	+
**CyEM-165**	GE581808	188	TC	10	182-194	4	3,004	0,393	0,667	-	-	-
CyEM-166*	GE605263	189	CT	10	-	\		\	\	+	+	+
**CyEM-167**	CL1848Contig1	189	AG	11	173-212	13	7,095	0,643	0,859	+	+	+
**CyEM-169**	GE610017	190	CTT	7	176-191	4	1,958	0,393	0,489	+	+	+
**CyEM-170**	GE598301	192	TGG	7	186-198	4	1,292	0,179	0,226	-	-	+
**CyEM-171**	GE610233	194	TCA	7	196-252	8	3,136	0,429	0,681	-	-	+
**CyEM-172**	CL5891Contig1	195	CT	11	394-432	13	4,532	0,393	0,779	+	+	+
**CyEM-173**	GE607339	196	GAC	7	187-204	6	2,777	0,593	0,640	+	+	+
**CyEM-174**	GE609927	196	CCA	7	109-209	7	3,503	0,538	0,714	+	+	+
**CyEM-175**	CL6045Contig1	197	CT	11	190-211	6	5,058	0,643	0,802	+	+	+
**CyEM-176**	GE604158	198	ACA	7	191-203	4	3,588	0,393	0,721	+	+	+
**CyEM-178**	GE612882	200	CAG	7	188-202	5	2,883	0,385	0,653	+	+	+
**CyEM-179**	GE601991	201	ATG	7	194-203	3	1,338	0,000	0,253	-	-	-
**CyEM-180**	CL431Contig1	201	CT	11	194-202	5	2,920	0,536	0,658	-	-	+
**CyEM-181**	GE577085	108	GT	11	96-111	8	4,599	0,593	0,783	+	+	+
**CyEM-182**	GE607197	202	TCA	7	192-225	9	6,453	0,393	0,845	+	-	+
**CyEM-183**	GE597664	98	CAA	7	91-103	5	3,174	0,786	0,685	+	+	+
**CyEM-185**	GE610261	208	GAA	7	183-296	12	6,258	0,741	0,840	+	+	+
**CyEM-186**	GE607652	209	CTC	7	198-216	7	4,695	0,536	0,787	-	-	-
**CyEM-187**	GE602677	210	AGC	7	255-261	3	1,075	0,071	0,070	-	-	-
**CyEM-188**	GE613233	210	GCA	7	194-225	10	6,288	0,538	0,841	+	+	+
**CyEM-189**	GE605002	215	ATC	7	211-220	4	2,877	0,571	0,652	+	+	+
**CyEM-190**	CL1046Contig1	242	CAG	7	235-250	6	3,530	0,630	0,717	+	+	+
**CyEM-193**	CL1609Contig2	246	ATC	7	254-268	6	2,256	0,571	0,557	+	+	+
**CyEM-195**	CL6448Contig1	249	ACA	7	397-420	7	3,778	0,357	0,735	+	+	+
**CyEM-196**	GE580410	250	CAT	7	238-267	8	3,919	0,231	0,745	+	+	+
**CyEM-197**	CL3269Contig1	250	TGA	7	245-274	7	3,496	0,370	0,714	-	+	-
**CyEM-199**	CL3496Contig1	251	ATC	7	246-260	8	6,426	0,679	0,844	+	+	+
**CyEM-200**	CL4126Contig1	237	CAT	7	217-259	6	3,471	0,519	0,712	+	+	+
**CyEM-201**	CL6303Contig1	252	GAA	7	225-251	6	2,521	0,214	0,603	+	+	-
**CyEM-202**	CL2754Contig1	105	CAG	7	92-106	5	4,084	0,630	0,755	+	+	+
**CyEM-203**	CL2776Contig1	253	ACC	7	325-330	3	2,074	0,333	0,518	-	-	+
**CyEM-204**	CL5986Contig1	253	TCT	7	242-263	7	3,769	0,321	0,735	-	+	-
**CyEM-205**	CL3033Contig1	237	CAC	7	230-239	4	3,073	0,462	0,675	+	+	+
**CyEM-207**	CL4470Contig1	257	CAC	7	240-268	10	6,231	0,741	0,840	+	+	+
**CyEM-208**	CL5699Contig1	257	AAG	7	254-256	2	1,080	0,000	0,074	-	-	-
**CyEM-209**	CL1652Contig1	188	ATC	7	180-194	5	1,576	0,143	0,365	+	+	+
**CyEM-210**	GE611460	260	TGA	8	253-267	6	3,446	0,478	0,710	+	+	+
**CyEM-211**	CL6394Contig1	120	GCA	7	114-123	4	2,116	0,370	0,527	+	+	+
**CyEM-212**	CL2349Contig1	262	ACC	7	262-268	3	1,124	0,038	0,110	-	-	-
**CyEM-213**	GE603351	263	TC	13	235-283	12	7,801	0,821	0,872	+	-	+
**CyEM-214**	CL1016Contig1	263	CAT	9	253-293	12	5,045	0,731	0,802	+	+	+
**CyEM-215**	CL6059Contig1	263	CAC	9	258-274	4	2,379	0,214	0,580	-	-	+
**CyEM-216**	GE578065	264	CAT	9	229-265	9	3,707	0,321	0,730	-	-	+
**CyEM-218**	GE611429	265	TC	13	350-370	9	5,580	0,536	0,821	+	+	+
**CyEM-219**	GE611316	267	CAC	8	256-278	6	2,925	0,464	0,658	+	+	-
**CyEM-220**	CL4549Contig1	274	CAT	9	261-276	6	4,326	0,704	0,769	+	+	+
**CyEM-221**	GE611385	275	TG	12	254-282	7	5,042	0,679	0,802	+	+	+
**CyEM-223**	CL5961Contig1	276	CAC	9	263-275	5	2,296	0,500	0,564	+	+	+
**CyEM-225**	GE580984	278	GAA	9	274-286	5	3,081	0,571	0,675	+	+	+
**CyEM-226**	GE611110	280	AGA	8	517-529	4	3,817	0,370	0,738	+	+	+
**CyEM-227**	CL5817Contig1	280	AAG	9	241-281	6	3,142	0,667	0,682	+	+	-
**CyEM-228**	CL4460Contig1	283	GAT	9	348-357	4	1,566	0,321	0,362	+	-	+
**CyEM-229**	GE577281	285	CT	13	270-294	10	6,222	0,786	0,839	+	+	+
**CyEM-230**	CL1174Contig1	285	CCA	9	271-293	6	3,159	0,462	0,683	+	+	+
CyEM-231*	CL548Contig1	286	AGA	9	-	\	\	\	\	+	+	+
**CyEM-232**	CL4621Contig1	287	CAG	9	273-287	6	4,915	0,679	0,797	+	+	+
**CyEM-233**	GE583211	288	AC	13	444-471	11	3,672	0,679	0,728	+	+	+
CyEM-234*	GE602543	310	TC	11	-	\	\	\	\	+	+	+
**CyEM-236**	CL923Contig1	315	CTT	8	401-433	6	3,729	0,667	0,732	+	+	+
**CyEM-237**	GE589921	316	CT	11	310-331	10	6,202	0,885	0,839	+	+	+
**CyEM-238**	CL1788Contig1	321	TC	12	304-334	9	2,904	0,296	0,656	-	-	-
**CyEM-240**	CL2307Contig1	324	AGC	8	303-333	8	5,063	0,630	0,802	+	+	+
**CyEM-241**	CL2526Contig1	325	CT	12	311-325	3	2,263	0,179	0,558	-	-	-
**CyEM-243**	CL5805Contig1	325	CT	12	309-338	10	4,472	0,667	0,776	+	+	+
**CyEM-244**	GE609380	326	TC	11	318-337	9	5,080	0,519	0,803	+	+	+
**CyEM-246**	GE604318	327	TG	11	320-354	8	3,117	0,520	0,679	+	+	+
**CyEM-247**	CL2951Contig1	327	CTG	8	316-339	7	3,815	0,500	0,738	+	+	+
**CyEM-248**	GE581850	330	AG	11	325-331	4	2,379	0,714	0,580	+	+	+
**CyEM-250**	CL3943Contig1	331	AG	10	321-334	5	3,516	0,321	0,716	+	+	+
**CyEM-253**	CL3338Contig1	142	CT	15	125-159	9	4,653	0,571	0,785	+	-	+
**CyEM-254**	CL3757Contig1	125	TC	12	108-141	15	7,682	0,654	0,870	+	+	+
**CyEM-256**	CL6387Contig1	338	TCA	8	323-350	7	4,148	0,464	0,759	-	+	-
**CyEM-259**	CL5381Contig1	197	CA	15	176-220	12	6,826	0,696	0,853	+	+	+
**CyEM-260**	CL2855Contig1	151	CCA	10	131-151	7	4,780	0,630	0,791	+	+	+
**CyEM-261**	GE613227	185	GGT	9	168-202	9	5,765	0,643	0,827	+	+	+
**CyEM-264**	CL3958Contig1	350	GAT	10	611-625	5	3,636	0,593	0,725	+	+	+
**CyEM-266**	GE598991	352	AG	14	343-359	8	5,985	0,571	0,833	+	+	+
**CyEM-272**	GE599540	356	TAC	9	348-357	4	3,333	0,600	0,700	+	+	+
CyEM-273*	GE599578	356	AAC	9	-	\		\	\	+	+	+
**CyEM-277**	CL2561Contig1	138	TC	14	116-157	13	5,911	0,783	0,831	+	+	+
**CyEM-278**	GE579023	391	CAT	10	376-399	5	3,057	0,259	0,673	+	-	+
**CyEM-279**	CL4781Contig1	106	TC	17	90-105	7	5,507	0,720	0,818	+	+	+
**CyEM-280**	GE591354	394	ATG	11	375-432	12	7,010	0,852	0,857	+	+	+
**CyEM-281**	GE610121	395	TC	15	372-412	9	5,302	0,778	0,811	+	-	+
**CyEM-282**	GE604802	399	GAT	11	380-401	5	4,085	0,423	0,755	+	+	+
**CyEM-284**	CL2318Contig1	111	AG	17	88-114	9	5,209	0,679	0,808	+	+	+
**CyEM-285**	CL4633Contig1	109	CT	16	91-129	13	6,284	0,667	0,841	+	-	+
**CyEM-286**	GE602088	405	TC	17	386-410	9	5,074	0,679	0,803	+	+	+
**CyEM-288**	GE595961	410	ATG	10	388-425	7	3,072	0,542	0,674	+	+	+
**CyEM-289**	GE580749	411	CTT	12	461-501	4	1,247	0,214	0,198	+	+	+
**CyEM-290**	GE583378	160	TC	15	151-174	9	5,383	0,500	0,814	+	-	-
**CyEM-291**	CL1901Contig1	147	TG	18	120-164	9	3,540	0,607	0,717	+	+	+
**CyEM-293**	CL3287Contig1	413	TC	20	384-410	9	4,717	0,440	0,788	+	+	+
**CyEM-294**	GE593962	231	GAT	11	216-232	7	4,556	0,407	0,781	+	+	+
**CyEM-295**	CL2047Contig1	414	AG	16	480-517	9	3,282	0,500	0,695	+	+	+
**CyEM-296**	GE602341	415	GAT	11	397-428	8	5,629	0,593	0,822	+	+	+
**CyEM-299**	CL6551Contig1	424	CAC	12	469-520	9	2,254	0,333	0,556	+	-	+
**CyEM-300**	GE610516	425	TC	15	405-432	7	3,885	0,308	0,743	+	+	+
**Average**						6,6	3,677	0,484	0,660			
**s.e**.						0,2	0,107	0,013	0,012			

Main information reported: locus name, unigene (for contigs-derived loci)/Accession number (for singletons-derived loci), expected size, perfect microsatellite motif, number of repeats, observed alleles range, number of observed alleles (n_o_), effective alleles (n_e_), observed heterozygosity (H_o_), polymorphic information content (PIC) and mapping utility for the three progenies ["Romanesco C3" × "Spinoso di Palermo" (GxG), "Romanesco C3" × "*altilis*41" (GxC) and "Romanesco C3" × "Creta-4" (GxW)]. Additional information are available in the *electronic supplementary material*. *Data showing a multi-locus amplification were excluded from the analysis.

**Figure 3:**
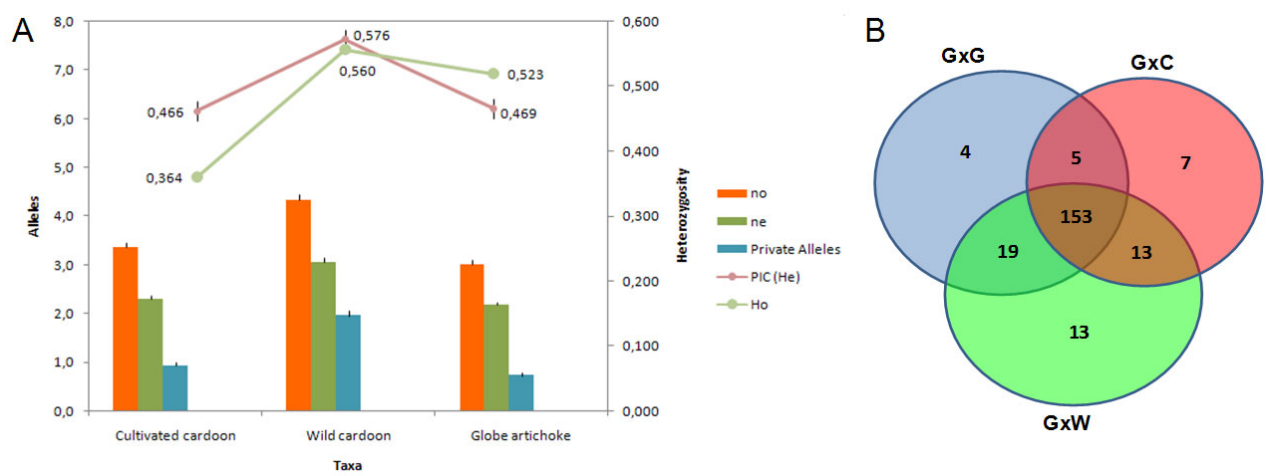
**Allelic diversity revealed by the set of EST microsatellite markers**. (A) Allelic patterns and the level of heterozygosity within each taxon. Observed (n_o_), effective (n_e_) and the number of taxon-specific alleles per marker are represented by bars. PIC and H_o _are indicated by points. (B) Markers showing segregation within the three mapping populations. GxG: within *scolymus*, GxC: *scolymus *× *altilis*, GxW: *scolymus *× *sylvestris*.

Allelic diversity was, as expected given the breeding history of these taxa [2,20,21], greater in the wild than in the two cultivated forms (Figure 3A). The frequency of taxon-specific alleles was two fold more in the wild cardoon, and the polymorphic information content (PIC) was higher in the wild cardoon (0.576 ± 0.015) than in either the globe artichoke (0.484 ± 0.013) or the cultivated cardoon (0.466 ± 0.015). The observed heterozygosity level (H_o_) was significantly less in the cultivated cardoon than in globe artichoke, presumably because the globe artichoke is primarily a vegetatively propagated plant, and thus able to maintain a high level of heterozygosity over time [20,21]; whereas cultivated cardoon is seed propagated and has been subjected to purifying selection aimed at increasing genetic uniformity for stabilizing its production. We previously developed three mapping population for the development of *C. cardunculus *genetic maps by crossing one globe artichoke non spiny genotype (common female parent) with a spiny genotype of globe artichoke or cultivated cardoon or wild cardoon. When the parents of the three mapping populations were tested with the set of microsatellites, 214 were informative in at least one combination, while 153 across all the three combinations, thus supplying landmarks for comparative mapping of phenotypic and quantitative trait loci (QTLs). As expected, the most polymorphic combination (198 microsatellites) was the one involving the cross between the most genetically divergent taxa: globe artichoke and wild cardoon (Figure 3B).

### Diversity analysis

**Figure 4:**
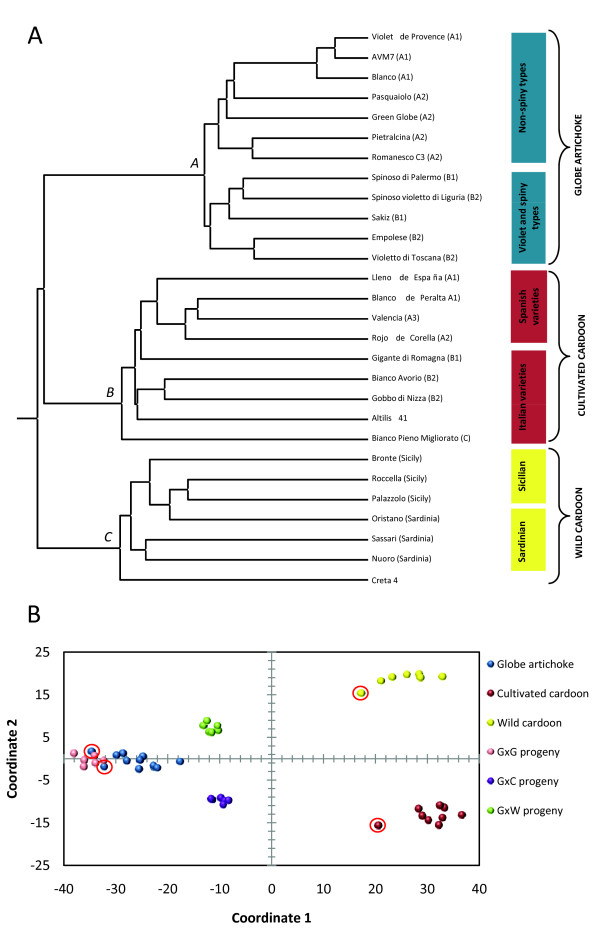
**Diversity analysis**. (A) A UPGMA dendogram based on 1,546 EST microsatellite alleles. The parentheses indicate the globe artichoke, cultivated cardoon and wild cardoon clusters defined by [20,21,26]. (B) Principal co-ordinate analysis based on the genetic distance matrix of 46 individuals, including the parents (red circles) and progeny of the three mapping populations GxG: within *scolymus*, GxC: *scolymus *× *altilis*, GxW: *scolymus *× *sylvestris*.

The informativeness of the newly developed EST microsatellites was comparable with that described for microsatellite markers in globe artichoke [13,22], sunflower [23,24] and lettuce [25]. A set of five EST microsatellites was sufficient to discriminate between each of the 28 members of the germplasm panel (e.g. CyEM-10, -37, -54, -105, -254). The inferred genetic relationships were in good concordance with those derived from AFLP profiling [20,21,26,27]. Thus, the globe artichoke accessions clustered with one another (Figure 4A, cluster A), but two sub-clusters, corresponding to the contrasting capitulum types (i.e. non spiny *vs*. violet, spiny types), could be recognised. The clade most closely related to the globe artichokes contained the cultivated cardoons (Figure 4A, cluster B), and among these, the most well differentiated accession was 'Bianco Pieno Migliorato', as previously observed [21]. The Spanish cultivated cardoon accessions were genetically very similar to one another. The wild cardoon accessions formed a discrete, but rather loose group (Figure 4A, cluster C). A principal co-ordinate analysis further illustrated the genetic relationships between members of the germplasm panel (Figure 4B). Axes 1 and 2 accounted for ~ 73% of the genetic variation, the former contributing ~ 47%, and the latter ~ 26%. Axis 1 distinguished the globe artichokes from the cultivated and wild cardoons, while Axis 2 separated the two cardoon taxa. As expected, F_1 _progenies mapped to intermediate positions with respect to those of their parents (Figure 4B).

### Distribution of microsatellite

**Figure 5:**
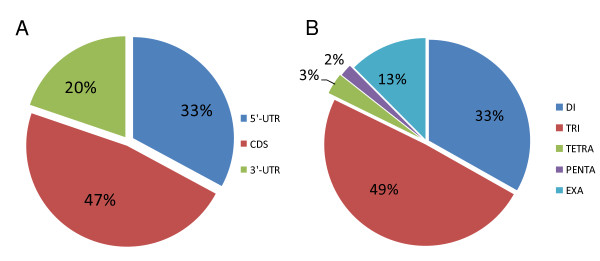
**Position and motifs of EST microsatellites**. (A) Distribution within specific regions of the unigenes. (B) Frequencies of repeat motifs within the unigene set.

**Figure 6:**
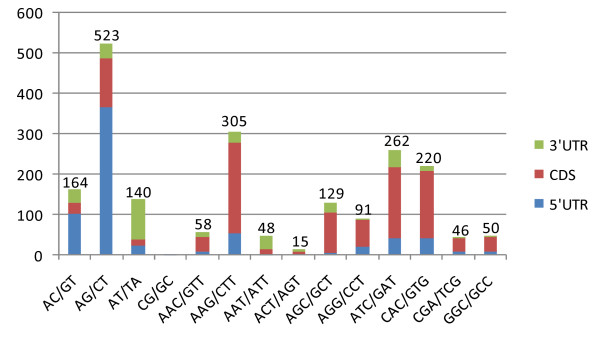
**Distribution of microsatellite classes. Di- and trinucleotide classes belonging to each unigene region (5'-UTR, coding sequence, 3-UTR)**.

Of the 4,219 microsatellites, trinucleotide motifs accounted for 49%, dinucleotides for 33%, hexanucleotides for 13%, tetranucleotides for 3% and pentanucleotides for 2% (Figure 5). Only ESTs (2,498 of the 3,308) having a non-ambiguous ortholog in *Arabidopsis thaliana *were taken forward for the purpose of annotation. The position of the microsatellite tract (5'-UTR or 3'-UTR or CDS) was derived from the BlastX result in conjunction with the ORF (Open Reading Frame)-Predictor algorithm [28]. About 33% of the microsatellites were present in the 5'-UTRs, ~ 20% in the 3'-UTRs and ~ 47% in the CDSs (Figure 5), similar to the relative frequencies in both the *A. thaliana *and rice genomes [15]. Most of the CDS microsatellites consisted of trinucleotides, while dinucleotides were the most abundant in the 5'-UTRs, and di- and trinucleotides were equally represented in the 3'-UTRs. Tetra- and pentanucleotide motifs were more frequent in the 3'-UTRs than in either the CDSs or the 5'-UTRs (data not shown). Trinucleotide (and hexanucleotide) motifs are expected to predominate in the population of CDS microsatellites, as variation in their repeat number is not associated with a frame shift event [29]. The most abundant dinucleotide repeat was AG/CT, followed by AC/GT, although AT/TA predominated in the 3'-UTRs. Among the trinucleotides, the most frequent was AAG/CTT, followed by ATC/GAT and CAC/GTG (Figure 6). This distribution is consistent with the situation in *A. thaliana *and *Brassica *spp. orthologs, in which a preference for AG/CT and AAG/CTT motifs has been identified in the 5'-UTRs, thought to be associated with the *cis*-acting regulation of transcription [30]. In the globe artichoke 5' UTRs, dinucleotide motifs were over-represented, with AG/CT being the most abundant (Figure 6), similar to the situation in the 5'-UTRs of many plant (both mono- and dicotyledonous species) genes [31,32], which has been reported to play a role in post-transcriptional gene regulation at the RNA level [33,34]. Dinucleotide motifs were also frequent in the 3'-UTRs, possibly because AT-rich elements are able to act as *cis *mediators of mRNA turnover [33]. Overall, present data confirm that homopurine/homopyrimidine repeats contribute markedly in 5'-UTR and CDS, as previously reported by Morgante *et al *[15].

### The function of genes containing microsatellites

Microsatellites within coding sequences can have a major effect on gene activity, since the expansion/contraction of triplets within the coding sequence alters the gene product, thereby sometimes causing a significant phenotypic change. In humans, the effects on phenotype due to the presence of SSRs in coding regions of genes playing key roles in neuronal disorders and cancer have been extensively studied [35]. Among the microsatellites in the globe artichoke transcriptome, the six most frequent amino acid stretches present in the CDS were poly-serine (94 unigenes), poly-aspartic acid (75 unigenes), poly-glutamic acid (57 unigenes), poly-lysine (46 unigenes), poly-glycine (45 unigenes) and poly-threonine (35 unigenes). It has been reported that particular amino acid repeats tend to be associated with specific classes of proteins [36]. Acidic and polar amino acid repeats have generally been associated with transcription factors and protein kinases, whereas serine repeats are common within membrane transporter proteins [37]. In the globe artichoke, poly-serine and poly-glycine stretches are particularly frequent in the CDS. Poly-serine linkers are common in eukaryotic genomes, and are thought to provide a flexible interdomain. They are frequently associated with modular proteins, and are involved in complex carbohydrate degradation [38] and the binding of proteins with extracellular matrix components, such as the laminin binding protein. Poly-glycine (also poly-asparagine and poly-proline) microsatellites may provide a domain for DNA binding or protein-protein interactions, and has been found to be necessary for chloroplast envelope targeting. Poly-glutamic and poly-aspartic acid tracts feature in many NLS (nuclear localisation signal) proteins [39], and it has been suggested that both basic karyophilic and acidic clusters can enhance their selective binding to transport machinery components [40]. Poly-glutamic acid stretches have also been implicated in transcription activation/de-activation [41-45], and microsatellite allelic variants of these genes have been identified as the genetic basis of a number of human diseases [46].

To support the occurrence of certain repeated motifs in the assembled unigenes we have exploited sequence alignment and gene ontology in order to annotate their functions and assess whether their motif type and position are preferentially associated with a particular gene ontology term.

In preparation, the set of globe artichoke unigenes was filtered to include only those with a BlastX E-value of < 1e^-29 ^when matched with the *A. thaliana *reference protein set. In all, 12,783 queries satisfied this criterion (Additional file 6: 12,783 globe artichoke unigenes annotation). The *A. thaliana *gene accession numbers were used to categorize the unigenes using TAIR gene ontology (data not shown). The GoStat2 web interface was then used to identify gene ontology categories which were over-represented. By comparing either the set of microsatellite-containing unigenes, or subsets of it (e.g.: genes including di- or trinucleotide motifs in their CDS or UTRs) with the complete set of annotated unigenes, it was possible to identify over-representation in gene ontology (GO) categories (Table 3). Microsatellites appeared to be over-represented in loci involved in certain biological processes and functions, while no significant association was found with GO cell components (data not shown).

**Table 3:** Functional enrichments.

					**CDS**						**5'-UTR**			
						
	**ID**	**Name**	**unigenes**	**All SSRs**	**All SSRs**	**TRI**	**DI**	**AAG_CTT**	**ATC_GAT**	**AG_CT**	**All SSRs**	**DI**	**TRI**	**AG_CT**
						
**Biological process**	GO:0006139	nucleic acid metabolic process	7,33	9,58	10,90	10,88	-	-	-	-	-	-	-	-
	GO:0019219	regulation nucleic acid metab. process	3,30	5,85	7,56	8,21	-	-	9,09	-	-	-	-	7,86
	GO:0006350	transcription	3,56	5,85	7,70	8,21	-	-	9,09	-	-	-	-	7,42
	GO:0006351	transcription, DNA-dependent	1,99	-	4,65	4,96	-	-	-	-	-	-	-	-
	GO:0006355	regulation of transcript., DNA-depend.	1,92	-	4,51	4,96	-	-	-	-	-	-	-	-
	GO:0010467	gene expression	8,12	-	11,19	11,64	-	-	-	-	-	-	-	-
	GO:0010468	regulation of gene expression	3,50	6,07	7,70	8,40	-	-	9,09	-	-	-	-	7,42
	GO:0016070	RNA metabolic process	3,51	-	5,96	6,11	-	-	-	-	-	-	-	-
	GO:0019222	regulation of metabolic process	3,62	6,36	7,85	8,59	-	-	9,09	-	-	6,83	-	7,86
	GO:0031323	regulation of cellular metabolic process	3,45	6,14	7,70	8,40	-	-	9,09	-	-	-	-	7,86
	GO:0032774	RNA biosynthetic process	1,99	-	4,65	4,96	-	-	-	-	-	-	-	-
	GO:0045449	regulation of transcription	3,29	5,77	7,56	8,21	-	-	9,09	-	-	-	-	7,42
	GO:0050789	regulation of biological process	4,17	6,94	8,58	9,16	-	-	-	-	-	-	-	-
	GO:0050794	regulation of cellular process	3,82	6,65	8,43	8,97	-	-	9,09	-	-	-	-	7,86
	GO:0065007	biological regulation	5,07	7,68	9,16	9,73	-	-	10,74	-	-	-	-	-
	GO:0006281	DNA repair	0,48	-	-	-	-	-	-	3,61	-	-	-	-
	GO:0002376	immune system process	0,32	-	-	-	2,73	-	-	-	-	-	-	-
	GO:0050896	response to stimulus	5,60	-	-	-	11,82	-	-	13,25	-	-	-	-
	GO:0006950	response to stress	2,71	-	-	-	-	-	-	8,43	-	-	-	-
	GO:0006974	response to DNA damage stimulus	0,48	-	-	-	-	-	-	3,61	-	-	-	-
	GO:0006855	multidrug transport	0,14	-	-	-	-	-	-	2,41	-	-	-	-
	GO:0009266	response to temperature stimulus	0,63	1,46	-	-	-	-	-	-	1,90	-	-	-
	GO:0009409	response to cold	0,38	-	-	-	-	-	-	-	1,14	-	-	-
	GO:0009628	response to abiotic stimulus	1,46	-	-	-	-	-	-	-	2,91	-	-	-
	GO:0009733	response to auxin stimulus	0,26	-	-	-	-	-	-	-	-	-	2,10	-
	GO:0009737	response to abscisic acid stimulus	0,27	-	-	-	-	-	-	-	-	1,71	-	1,75
	GO:0009738	abscisic acid mediated signaling	0,11	-	-	-	-	-	-	-	-	1,02	-	1,31
	GO:0065004	protein-DNA complex assembly	0,19	-	-	-	-	2,17	-	-	-	-	-	-
	GO:0046483	heterocycle metabolic process	0,75	-	-	-	-	3,62	-	-	-	-	-	-
**Molecular function**	GO:0003676	nucleic acid binding	8,89	13,67	17,01	-	-	18,12	-	18,07	-	-	-	-
	GO:0003677	DNA binding	5,05	8,92	11,34	-	-	-	13,22	-	-	-	-	10,04
	GO:0003700	transcription factor activity	3,34	6,51	8,14	-	-	-	9,09	-	-	6,48	-	8,30
	GO:0008270	zinc ion binding	3,18	4,39	-	-	-	-	-	-	-	-	-	-
	GO:0043169	cation binding	4,89	6,65	-	-	-	-	-	-	-	-	-	-
	GO:0016667	oxidoreduct. activ. on SH group of donors	0,13	-	-	-	-	-	-	2,41	-	-	-	-
	GO:0015297	antiporter activity	0,45	-	-	-	-	-	-	3,61	-	-	-	-
	GO:0051219	phosphoprotein binding	0,11	-	-	-	-	-	-	-	0,63	1,71	-	1,75
	GO:0004721	phosphoprotein phosphatase activity	0,93	-	-	-	-	-	-	-	2,02	-	-	-
	GO:0005544	Ca-dependent phospholipid binding	0,03	-	-	-	-	-	-	-	-	-	1,40	-
	GO:0015071	protein serine/threonine phosphatase activity	0,45	-	-	-	-	-	-	-	-	-	-	2,18

GO terms statistically enriched (showed in percentage) for specific SSRs subsets. Fisher's exact test was performed between each SSRs subset versus the whole unigenes categorization; only significant over-represented subset are reported (p < 0,01). Analysis is displayed referring to "biological process" and "molecular function" classifications; "cellular component" is not reported due to the absence of particular enriched subsets.

Most of the unigenes containing trinucleotide motifs in their CDS were associated with nucleic acid metabolic processes (GO:0006139), transcription (GO:0006350) and the regulation of transcription (GO:0006355), consistent with the encoding by the GAT trinucleotide of aspartic acid, since stretches of this residue are characteristic of 'karyophilic' acidic clusters in NLS (nuclear localization signal) proteins. Similarly, the AAG/TTC motif, which occurred frequently in the CDS, encodes poly-glutamate, which is thought to be involved in both protein-DNA complex assembly (GO:0065004) and heterocyclic metabolic processes (GO:0046483). Unigenes carrying dinucleotide motifs in their CDS were found to be specifically associated with the response to stimulus (GO:0050896). The AG/CT repeats in the CDSs were over-represented among genes responding to stress (GO:0006950), involved in DNA repair (GO:0006281) and in nucleic acid binding (GO:0003676). This is consistent with the presence of domains involved in protein-RNA/protein-protein sticky interactions.

The commonest microsatellite motifs occurring in 5'-UTR of unigenes were dinucleotide repeats (mostly AG/CT). These unigenes were associated with nucleic acid metabolism (GO:0006139), the regulation of gene expression (GO:0010468), transcription (GO:0006350) and the regulation of transcription (GO:0006355). AG/CT repeats were also over-represented in genes involved in the response to ABA (GO:0009737 and GO:0009738). Moreover, *trans-acting *elements (GO:0003700: "transcription factor activity"), which show an over-representation of trinucleotidic (ATC/GAT) in their CDSs, were also frequently enriched in their 5'UTRs by AG/CT motifs, suggesting a cascade of signal transmission. Trinucleotide motifs were not common in the 5'-UTRs, except in genes involved in the response to auxin stimulus (GO:0009733).

## Conclusion

We have demonstrated here the utility of a set of *de novo *globe artichoke EST-based microsatellite markers for the definition of genetic diversity, phylogeny and genetic mapping. Since EST microsatellites lie within expressed sequences, they have the potential to represent perfect markers for genes underlying phenotypic variation. Most of these assays are fully transferable to other *C. cardunculus *taxa, providing anchor points for the integration of taxon-specific genetic maps. The functional annotation of these EST sequences increases their utility as a source of gene-based markers for the study of synteny and other applications.

## Methods

### EST microsatellites discovery and primer design

A collection of 36,321 EST, generated from the 'Green Globe' variety of *C. cardunculus *var. *scolymus*, as part of the output of the Compositae Genome Project , was downloaded from the NCBI database . To generate a set of unique assemblies, the sequences were first trimmed to remove any remaining vector fragments and polyA tails, using the perl script SeqCleaner, and assembled adopting a second perl script, TGICL, employing the following parameters: p = 95 (identity percentage), l = 40 (minimum overlap length), v = 10 (maximum length of unmatched overhangs); the maximum mismatch overhang was set to 10 bp, since the sequences had already been purged of vector stretches and polyA tails. The two scripts are available at . The unigene set was then searched for perfect microsatellite sequences using a modified SSRIT perl script [47], with the minimum number of dinucleotides set as five, of tri-, tetra- and penta-nucleotides set as four, and of hexanucleotides as three. A sample of 300 non redundant microsatellite-containing sequences, selected to include the longer microsatellite motifs, was taken forward for PCR screening. Primer design was carried out using BatchPrimer3  with an optimal GC content of 50%, a maximum melting temperature difference of 3°C, variable amplicon size (to allow multiplexing), and all other parameters set to default values. The *de novo *microsatellite markers were prefixed with 'CyEM' (Cynara Expressed Microsatellite) and numbered sequentially.

### Plant materials and genomic DNA isolation

DNA was extracted from young leaves following a modified CTAB method [48]. The primers were used to amplify genomic DNA template extracted from a germplasm panel consisting of twelve globe artichoke genotypes, representative of crops grown in the Mediterranean Basin [20]; nine cultivated cardoon genotypes, representative of both the Spanish and Italian gene pools [21]; and seven wild cardoon genotypes sampled from both Sicily and Sardinia [26]. Full genotypes details are reported in Table 1. The set also included DNA of the four parents of three established mapping populations, i.e. two globe artichoke accessions ['Romanesco C3' (C3) and 'Spinoso di Palermo' (SP)], one cultivated cardoon ('Altilis 41') and one wild cardoon ('Creta 4'); furthermore six F1 individuals from each of the segregating populations (C3 × SP, C3 × Altilis 41 and C3 × Creta 4) were included in the analyses.

### Genotyping and diversity analysis

Primer pairs CyEM-001 to CyEM-300 (Additional file 4: primer pairs designed) were tested for their informativeness on the germplasm panel. Amplification was carried out in 10 μl reactions containing 7 ng template DNA, 1× PCR Buffer (Qiagen Inc., Venlo, Netherlands), 1.0 mM MgCl_2_, 0.5 U Taq DNA polymerase (Qiagen), 40 nM 5'-labelled (FAM, HEX or TAMRA) forward primer, 40 nM unlabelled reverse primer and 0.2 mM dNTPs. A touchdown cycling regime was applied, consisting of 1 cycle at 94°C for 150 sec, 9 cycles at 94°C for 30 sec, 63°C for 30 sec (-0,7°C/cycle) and 72°C for 60 sec, then 30 cycles at 94°C for 30 sec, 57°C for 30 sec and 72°C for 60 sec, followed by a final extension at 72°C for 5 min.

Weakly amplified reactions were re-run using 1.5 mM MgCl_2 _and applying a final annealing temperature of 55°C. Amplicons were separated on an ABI3730 capillary DNA sequencer (Applied Biosystem Inc., Foster City, CA, USA). Internal ROX-labelled GS500 size standards were included in each capillary. Fragment data were analysed using GeneMapper v3.5 software (Applied Biosystems). The genotypic data were analysed using the GenAlex Excel package [49]. Genetic diversity was calculated separately for the globe artichoke, cultivated cardoon and wild cardoon genotypes on the basis of (1) the mean number of alleles observed per locus (n_o_), (2) the effective number of alleles per locus (n_e_) as predicted by 1/Σp_i_^2 ^where p_i _is the frequency of the *i*^th ^allele at the locus, (3) the mean observed heterozygosity (H_o_), and (4) the polymorphic information content (PIC), estimated following [49]. A co-phenetic distance matrix for co-dominant markers was generated as described by Smouse and Peakall [50] and used to construct a UPGMA-based dendrogram [51] by means of NTSYS software package v2.10 [52]. Principal co-ordinate analysis was based on the distance matrix, with data standardization provided by the GenAlex package.

### Annotation of the unigene set

The unigene set was aligned by a BlastX [53] search against the *A. thaliana *reference proteins database (NCBI), applying an E-value threshold of e-^29^. The location within the gene sequence of the microsatellite (5'-UTR, CDS or 3'-UTR) was inferred from this alignment, while ORF-Predictor [28] was used to predict the position of the stop codon. The frequencies of peptide repeat tracts within the CDS were used to identify any over-representation of particular triplets. For this purpose, the unigenes were divided into ten subgroups on the basis of the identity of the trinucleotide motif present in the CDS. Each subgroup was then subjected to an analysis based on the ORF-Predictor algorithm, considering only the positive reading frames (+1, +2, +3), since the sequenced transcripts were originally directionally cloned. All the unigenes were assigned a function based on the GeneOntology tool , using the *A. thaliana *orthologs (from BlastX output) as input (using AGI codes from TAIR). Enrichment within the GO hierarchical levels by mean of different subset of unigenes was estimated using the GoStat2 interface  based on Fisher's exact test [54], adopting a threshold p-value of 0.01 and considering terms starting from the 3rd hierarchical level of the DAG (directed acyclic graph; Table 3).

## Authors' contributions

SL and SK planned and supervised the work. DS were responsible for the *in silico *analysis of the EST sequence data, primer design and amplification; AA and EP selected the constitution of the *C. cardunculus *gerplasm panel; DS, AA, EP and CT analysed the data. All the authors contributed to the final version of the manuscript.

## Supplementary Material

Additional file 1 **EST assembly**. The data provided represent the list of 6,621 assembled globe artichoke contigs derived from 23,871 ESTs. Click here for file

Additional file 2 **19,055 unigenes**. The data provided represent the fasta sequences of the assembled unigenes (contigs and singletons). Click here for file

Additional file 3 **ACE assembly file (EagleView available at **. Contig representation file parsed from TGICL output file by a customised PERL script. Click here for file

Additional file 4 **primer pairs designed**. The data provided represent the list of the 2,311 designed primer pairs and loci/SSRs description. Click here for file

Additional file 5 **full statistics on 300 SSR-containing loci**. The data provided represent the list of 300 selected SSR-containing loci, their allele statistics, polymorphism information, repetitive motif position and gene annotation. Click here for file

Additional file 6 **12,783 globe artichoke unigenes annotation**. The data provided represent the list of 12,783 globe artichoke unigenes annotation using BlastX (e-value threshold < 1e^-29^) on Arabidopsis reference protein database (June, 2008). Click here for file
