# Supplementary material for: Ontology and diversity of transcript-associated microsatellites mined from a globe artichoke EST database
Source: BMC Genomics. 2009 Sep 28;10:454. doi: 10.1186/1471-2164-10-454 (PMC2760586; doi:10.1186/1471-2164-10-454)
Supplement: Additional file 3 — ACE assembly file (EagleView available at . Contig representation file parsed from TGICL output file by a customised PERL script. [file 1471-2164-10-454-S3.ZIP › Scaglione_et_al.doc]

**Ontology and diversity of transcript-associated microsatellites mined from a globe artichoke EST database**

Davide Scaglione^1^, Alberto Acquadro^1^, Ezio Portis^1^, Chris Taylor^2^, Sergio Lanteri^1*^, Steven J. Knapp^2^

^1^Di.Va.P.R.A. Plant Genetics and Breeding, University of Turin, via L. da Vinci 44, 10095 Grugliasco (Turin), Italy

^2^Institute for Plant Breeding, Genetics, and Genomics, University of Georgia, 111 Riverbend Rd., 30602 Athens, Georgia (U.S.A.).

*corresponding author

Email addresses:

DS: [davide.scaglione@unito.it](mailto:davide.scaglione@unito.it)

AA: [alberto.acquadro@unito.it](mailto:alberto.acquadro@unito.it)

EP: [ezio.portis@unito.it](mailto:ezio.portis@unito.it)

CT: [taylor75@uga.edu](mailto:taylor75@uga.edu)

SL: [sergio.lanteri@unito.it](mailto:sergio.lanteri@unito.it)

SK: [sjknapp@uga.edu](mailto:sjknapp@uga.edu)

**Abstract**

**Background:** Globe artichoke (*Cynara cardunculus* var. *scolymus* L.), a member of the Asteraceae (=Compositae) family, is an important crop for the Mediterranean basin agricultural economy. In spite of its commercial relevance and nutraceutical and pharmaceutical properties, its genome structure has been elucidated to a limited extent. Microsatellite (SSR = simple sequence repeat) markers are known to be a reliable tool in genomic studies. Based on the ESTs (expressed sequence tags) of globe artichoke, recently made available by the Composite Genome Project (CGP), we developed a set of SSR markers.

**Results:** Using a custom bioinformatic pipeline, 36,321 Sanger ESTs were trimmed, assembled into 19,055 unigenes (6,621 contigs and 12,434), annotated, and mined for perfect SSRs. BLASTX alignment against *Arabidopsis* reference proteins facilitated functional annotation of 12,321 *Cynara* *cardunculus* unigenes. We identified 4,219 perfect repeats in 3,308 unigenes (1 SSR per 3.6 kbp), designed genotyping primers for 2,311 SSRs with sufficient flanking sequences for primer placement, and developed and tested genotyping primers for 300 EST-SSRs. The latter were screened for allele length polymorphisms among 28 globe artichoke and wild and cultivated cardoon accessions. Of the 300 EST-SSR markers tested, 236 consistently amplified alleles of the predicted lengths, produced high-resolution genotypes, and were polymorphic among wild and cultivated *C.* *cardunculus* accessions. Polymorphic information content (PIC) ranged from 0.04 to 0.90 among markers and the mean PIC was 0.66 across markers and accessions. Moreover, 176 to 198 of the EST-SSR markers (75 to 84%) were polymorphic in three *Cynara* *cardunculus* mapping populations.

**Conclusion:** SSRs mined from a globe artichoke EST database yielded numerous highly polymorphic SSR genotyping assays for phylogenetic, marker-assisted breeding, transcript mapping, and other applications in *Cynara cardunculus*.

**Background**

*Cynara cardunculus* is a species in the *Asteraceae* (= *Compositae*) family, originating in the Mediterranean area [1]. It includes three taxa: the globe artichoke (var. *scolymus* L.), the cultivated cardoon (var. *altilis* DC), and their progenitor, the wild cardoon [var. *sylvestris* (Lamk) Fiori]. Globe artichoke is an important component of the Mediterranean diet, but today is widely cultivated all over the world; the edible part of the plant are the immature inflorescences, referred as *capitula* or heads [2]. *C. cardunculus* leaf extracts are rich in phytochemicals (e.g., quercetin, rutin, luteolin, gallic acids, di-caffeoylchinic acids, and sesquiterpene lactones) which have anti-oxidative and anti-carcinogenic activity, inhibit cholesterol biosynthesis, and enhance lipid metabolism [3-7]. Halvorsen *et al*. [8] ranked globe artichoke fourth for antioxidant content per serving among vegetables. Furthermore, roots are a source of inulin, which acts as an enhancer of human intestinal flora [9, 10].

In spite of the economic importance, globe artichoke improvement through breeding has been rather limited and its genome organization remains largely unexplored; thus a better knowledge of its genome is essential to move to a marker assisted breeding.

Molecular maps of globe artichoke, based on a two-way pseudo-testcross strategy, have recently been developed [11-13]. However new molecular tools have to be made available to better establish a framework of linkage relationships allowing the identification and localization of genes controlling yield traits, resistance to pathogens, as well as the production of metabolites of nutraceutical and pharmaceutical interest. In this regard, microsatellites (SSR = simple sequence repeat) are of particular interest because they are abundant, co-dominant, hypervariable [14-18], and suitable for high-throughput PCR-based platforms.

It was long assumed that SSRs were primarily associated with non-coding DNA, but it has now become clear that they are more frequent in transcribed than non-transcribed sequences and equally frequent in the transcriptomes of plants with dramatically different nuclear DNA contents [19]. A survey of EST databases therefore represents one of the most convenient approaches to mine sequences harbouring SSRs, with the aim to develop highly polymorphic gene-based markers which might be also exploited as COS (conserved orthologous set) markers for genomic comparative analysis.

Here we report the *in silico* isolation of EST-SSRs using a unigene set built up from the globe artichoke EST database deposited in GenBank by the Compositae Genome Project (CGP). A subset of informative SSR markers were evaluated for their utility for genomic and mapping studies. Furthermore, we performed a comprehensive functional annotation, inferred from sequence alignment (ISA), as well as a gene ontology categorisation inferred from sequence orthology (ISO) of the SSR-containing unigenes. At last we assessed whether motif type and relative position within CDS/UTR may be preferentially associated with a particular gene ontology term.

**Results and discussion**

**EST-SSR discovery, frequency and primer design**

*Cynara cardunculus* ESTs were trimmed, assembled, and annotated using a custom bioinformatic pipeline (Figure 1). The ESTs assembled into 19,055 unigenes (6,621 contigs + 12,434 singlestons) spanning 15 Mbp of the *C.* *cardunculus* transcriptome. The transcript assembly and unigene consensus sequences have been supplied as electronic supplementary materials (See Additional file 1,2,3: EST assembly, 19055 unigenes, ACE assembly file). The mean unigene length was 786 ± 1.7 bp, GC content ranged from 24.7 to 67.9%, mean GC content was 43.9 ± 0.03% (Figure 2), and the mean ambiguity code ratio was 0.51 ± 0.01 among unigenes. When the *C.* *cardunculus* EST database was mined for uninterrupted (perfect) dinucleotide to hexanucleotide repeats with a minimum of *n* = 4 tandem repeats, 4,219 were discovered in 3,308 unigenes. The mean SSR density was one per 3.6 kb. Comparable SSR frequencies and densities have been discovered in the transcriptomes of sunflower (*Helianthus annuus* L.) and lettuce (*Lactuca sativa* L.), other species in the Compositae [18, 20, 21].

We only screened for perfect repeats because they tend to be more prone to strand slippage and, consequently, to be more mutable and polymorphic than interrupted tandem repeats [22]. Furthermore, the number of perfect repeats identified in the *C. cardunculus* EST database was more than sufficient for developing several hundred high quality SSR genotyping assays. We designed genotyping primers for 2,311 perfect SSRs in 1,974 unigenes, the number with sequences of sufficient length and quality for placing suitable primers flanking the targeted SSRs (Additional File 4: primer pairs designed).

***Cynara cardunculus* EST-SSR marker development and allelic diversity**

We sampled a broad spectrum of repeat types and lengths for EST-SSR marker development and analysis. SSR genotyping primers were developed for 300 transcript-associated SSRs ranging in length from 10 to 83 bp. Target amplicon lengths ranged from 98 to 456 bp and were selected for a uniform distribution across the length range to maximize multiplex density on capillary genotyping platforms. The 300 SSR genotyping primers were screened for positive high-quality amplification among 12 globe artichoke, eight cultivated cardoon, and seven wild cardoon accessions (Table 1). Of the 300 EST-SSR markers, 238 (79.3%) amplified alleles and 236 were polymorphic among *taxa* and accessions and 215, 216, and 223 were polymorphic among globe artichoke, cultivated cardoon, and wild cardoon accessions, respectively. Specific characteristics of the EST-SSR markers, allele length ranges, and genetic diversity statistics are summarized for analyses across *C. cardunculus* in Table 2 and within individual *taxa* in Additional File 5 (Full statistics on 300 SSR-containing loci). Collectively, 1,546 alleles were amplified by 238 EST-SSR markers with a minimum, mean, and maximum of one, 3.8, and 15 alleles per locus, respectively. The widest allele length range (196 to 252 bp) was observed for an EST-SSR markers targeting a [TCA]_7_ repeat (CyEM-171). SSR alleles of the predicted length were amplified by 85% of the markers, whereas 12.2% amplified alleles longer than predicted. SSR genotyping primers for the latter presumably either spanned introns or insertions. The allele length range was significantly wider for wild cardoon (17.1 ± 1.0 bp) than globe artichoke (13.6 ± 0.8 bp) or cultivated cardoon (13.7 ± 0.7 bp).

SSR allelic diversity was greater in wild than cultivated *C. cardunculus* accessions (Figure 3a). The number of taxon-specific alleles was two-fold greater in wild cardoon than globe artichoke and cultivated cardoon, as predicted from the domestication and breeding history of these *taxa* [20-22]. The informativeness of EST-SSRs was greater in wild cardoon (average PIC value: 0.576 ± 0.015) than globe artichoke (0.484 ± 0.013) and cultivated cardoon (0.466 ± 0.015). Observed heterozygosity (H_o_) was significantly lower in seed-propagated cultivated cardoon than clonally propagated globe artichoke, a pattern produced by selection for increased uniformity in seed-propagated open-pollinated cultivars of cultivated cardoon versus the maintenance of heterozygosity by clonal propagation of heterotic, outbreeding individuals in globe artichoke [25, 31]. Collectively, 214 of the EST-SSR markers (80%) were polymorphic in one or more of three mapping populations developed from intra- and inter-taxa hybrids^__^198 were polymorphic in the globe artichoke x wild cardoon mapping population, the most polymorphic of the three (Figure 3b). Moreover, 153 were polymorphic across populations and supply landmarks for comparative mapping of phenotypic and quantitative trait loci

**Diversity analysis**

The informativeness of the EST-SSRs was comparable with the one previously found for microsatellite markers in globe artichoke [12, 23], sunflower [24, 25] and lettuce [26]. A minimal panel of five EST-SSRs markers uniquely identifies each of the 28 genotypes (e.g. CyEM-10, -37, -54, -105, -254). Despite the possibility of homoplasy (identical allele sizes may not be identical by descent), the genetic relationships inferred through SSR genotyping are in good agreement with those previously detected from AFLP profiling [20, 21, 27]. All the globe artichoke genotypes clustered together (Figure 4A, Cluster *A*) and two sub-clusters, corresponding to different head morphology characteristics, were recognised: the non spiny types being clearly separated from the violet and the spiny ones. The cluster closest to the globe artichoke clade included the cultivated cardoon genotypes (Figure 4A, Cluster *B*); among them the ‘Bianco Pieno Migliorato’ was the most genetically differentiated while, as previously observed [21], the Spanish accessions were characterized by an higher genetic similarity. As expected, the wild cardoon genotypes grouped together although to a lesser extent of genetic similarity compared with cultivated *taxa* (Figure 4A, Cluster *C*).

The principal co-ordinate (PCO) analysis illustrates the genetic relationships between the members of the genotype set (Figure 4B). The first two principal co-ordinates accounted for ~73% of the overall genetic variation, with each in turn contributing 47%, and 26%. The first co-ordinate distinguished the globe artichoke genotypes from cultivated and wild cardoon, while the second allowed the separation of each *taxon*. As expected each F_1_ progeny was located in an intermediate positions with respect to their parental genotypes (Figure 4B).

**Microsatellite distribution among unigenes**

Among the 4219 SSR-containing unigenes, the trinucleotidic motifs accounted for 49% followed by di- (33%), hexa- (13%), tetra (3%) and penta- (2%) (Figure 5). This set was filtered using BlastX, and only the sequences having a non ambiguous ortholog in *Arabidopsis* (2,498) were used for the following analysis. The microsatellite position within transcripts (5'-UTR, CDS and 3'-UTR) was automatically derived analysing BlastX generated data, in combination with ORF-Predictor algorithm.

Thirty-three percent of the SSRs were present in 5'-UTR, 20% in 3'-UTR and 47% in CDS, (Figure 5) confirming data from *A. thaliana* and *Oriza sativa* [19]. In CDS the majority of Most repeats in CDSs were tri-nucleotides, while in 5'-UTR dinucleotide were the most abundant, and in 3'-UTR both di- and tri-nucleotidic were equally represented. Tetranucleotide as well as pentanucleotide motifs were relatively more frequent in 3'-UTR than CDS and 5’-UTR (data not shown). This trend was expected since trinucleotides (as well as exanucleotides) are positively selected inside coding regions because of the characteristic to avoid frame shift events [28].

Among dinucleotide motifs, the most abundant was AG/CT (74% in 5'-UTR, 76% in CDS, Figure 6), followed by AC/GT (20,9% in 5'-UTR and 16,4% in CDS), while AT/TA was predominant in 3'-UTR (60%). The most frequent trinucleotidic motif was AAG/CTT (28% in 5'-UTR and 26% in CDS) followed by both ATC/GAT and CAC/GTG, the percentage of which was about 20% in CDS. This distribution supports a previous analysis which highlighted the preferential conservation of AG/CT and AAG/CTT motifs in the 5’-UTR of *A. thaliana* and *Brassica* ortholog genes, presumably involved as *cis*-acting elements in transcriptional or post-trascriptional regulation [29], and justify our investigation on the potential association between these subsets of microsatellite and GO terms (see below).

Microsatellite within coding sequences can be very critical for normal gene activity since variation due to expansions/contractions of coding triplets directly affects the corresponding gene products and might cause phenotypic changes. In humans, the effects on phenotype due to the presence of SSRs in coding regions of genes playing key roles in neuronal disorders and cancer have been extensively studied [30]. In the analysed globe artichoke EST SSR set, the six most frequent types of amino acid stretches in CDS were: poly-serine within 94 unigenes (coded by CTT and ATC codons); poly-aspartic acid within 75 unigenes (GAT); poly-glutamic acid within 57 unigenes (AAG); poly-lysine within 46 unigenes (AAG); poly-glycine within 45 unigenes (GTG); poly-threonine within 35 unigenes (CAC). It has been reported that different amino acid repeats are concentrated in different classes of proteins. Marked bias in favour of certain amino acids in different proteins or cell locations showed that triplet repeats in ORFs were not random with respect to the ORFs and DNA strands [31]. Acidic and polar amino acid repeats are generally associated with transcription factors and protein kinases, whereas serine repeats are significantly associated with membrane transporter proteins [32].

In globe artichoke polyserine and polyglycine stretches were found over-represented in CDS. A polyserine linker (PSL) is a flexible interdomain, common among eukaryotes, which has been found in modular proteins, and it is frequently involved in complex carbohydrate degradation [33] or in the binding of proteins with extracellular matrix components (e.g.: laminin binding protein). Polyglycine **(**as well as polyasparagine or polyproline) coding SSRs may provide a domain for DNA binding or protein-protein interaction and has been found to be necessary for chloroplast envelope targeting. The polyglutamic and polyaspartic acid stretches were also found over represented in the SSR filtered unigene. It is noteworthy that polyglutamic and polyaspartic acid tracts are features of many nuclear proteins [34]. It has been suggested that ‘‘karyophilic’’ acidic clusters in NLS (nuclear localization signal) proteins and mediates their selective binding to proteins of the transport machinery [35]. Poly glutamic acid stretches are also known to be involved in the activation/de-activation of transcription [17, 36-39], and allelic variants in such genic SSRs have been implicated as the genetic determinants of a number of human diseases [40].

In globe artichoke the 5' UTR dinucleotidic motifs were over represented (Figure 6) and AG/CT was the most abundant. This is in agreement with data available for the 5’-UTR of many monocot and dicot genes [41, 42], which has been reported to play a role in post-transcriptional gene regulation at the RNA level [43-45]. Dinucleotidic motifs were also frequent in the 3'-UTR, and the AT/TA was the most over represented. Interestingly, AT-rich elements were found to be a key cis-element in 3'-UTR, mediating the mRNA turnover [43]. On the whole the present data confirm that homopurine/homopyrimidine repeats contribute markedly in 5'-UTR and CDS, as previously reported by Morgante *et al.* [19].

**Annotation, categorization and functional relevance**

Globe artichoke unigenes were annotated considering only the unigene hits with e-value below to 1e^-29^, and 12783 queries reached this requirement (Additional file 6: 19055 globe artichoke unigenes annotation). The *Arabidopsis thaliana* accession numbers resulting from this annotation were used to create a categorization of unigenes by TAIR gene onthology (data not shown). The GoStat web interface allowed to determine over-represented gene ontology categories for biological processes/molecular functions. By computing 2497 SSR-containing unigenes or the splitted motif subsets (e.g.: di-, tri-, in CDS or UTR respectively) versus the whole annotated unigene set (12783 sequences), we discovered significant enrichments for 40 GO categories, starting from the 3^th^ hierarchical level of the DAG (directed acyclic graph; Table 3). Interestingly, SSRs were over-represented in loci involved in some specific biological processes and functions, while in cell components were found to have a less significant association (data not shown).

Overall, unigenes carrying trinucleotide motifs in CDSs (mostly ATC/GAT) are presumably involved in “nucleic acid metabolic process” (GO:0006139), “transcription” (GO:0006350) and “regulation of transcription” (GO:0006355). This is consistent with the fact that GAT repetitions code for poly-aspartic acid stretches found in ‘‘karyophilic’’ acidic clusters in NLS-proteins. AAG/TTC, an over-represented motif in CDSs coding for poly glutamate, appears to be involved in “protein-DNA complex assembly” (GO:0065004) as well as “hetero-cycle metabolic" processes (GO:0046483).

Unigenes carrying dinucleotide motifs in CDSs were found to be specifically associated with “response to stimulus” (GO:0050896), Interestingly, AG/CT repeats were over-represented in response to stress” (GO:0006950), and “DNA repair” (GO:0006281) GO biological processes, and in “nucleic acid binding” (GO:0003676) GO molecular functions. This is consistent with the presence of domains involved in protein-RNA/protein-protein sticky interactions.

As regard SSRs in 5’-UTR of unigenes, dinucleotidic repeats (mostly AG/CT) were over represented in “nucleic acid metabolic process” (GO:0006139), “regulation of gene expression” (GO:0010468), “transcription” (GO:0006350) and “regulation of transcription” (GO:0006355); besides, AG/CT repeats were over-represented in “response to ABA” (GO:0009737 and GO:0009738) GO categories. Interestingly, *trans-acting elements* like “transcription factor activity” (GO:0003700), which show an over-representation of trinucleotidic (ATC/GAT) in their CDSs, were also frequently enriched in 5’UTR by AG/CT motifs (“regulation in the regulation”), suggesting a cascade of signal transmission. Enrichments for trinucleotidic motifs in 5’-UTR, except for the “response to auxin stimulus” (GO:0009733), were absent. Data for non statistically significant subsets comparison were not showed.

**Conclusion**

Our results demonstrate that the newly developed set of *in silico* mined EST-SSR markers for globe artichoke is highly informative for both diversity, phylogenetic and mapping studies. Since EST-SSRs lie within expressed sequences, they have the potential to serve as perfect markers for genes which determine variation in phenotype. Most of them are transferable to other *C. cardunculus* *taxa*, providing anchoring points for future integration of genetic maps under construction. As SSR-containing loci were functionally annotated using *A.* *thaliana* orthologous unigenes, they represent a very promising source of gene based molecular markers for studies of synteny.

**Methods**

**EST-SSRs mining and primers design**

A collection of 36321 EST from *C. cardunculus* var. *scolymus*, developed inside the CPG (*Compositae Genome Project*) by Tangs *et al.* (2008, unpublished), was downloaded from NCBI database ([http://www.ncbi.nlm.nih.gov](http://www.ncbi.nlm.nih.gov/)). In order to generate unique assemblies, the first step consisted in sequences trimming for vector fragments or polyA tails with SeqCleaner perl script (<http://compbio.dfci.harvard.edu/tgi/software/>). To generate unique assemblies, another perl script, TGICL, was run with the following parameters: -p 95 (indentity percentage), -l 40 (minimum overlap length), -v 10 (maximum length of unmatched overhangs). The maximum mismatched overhangs was changed to 10bp assuming the sequences as already cleaned from vector stretches and polyA tails: these settings were adopted to avoid clustering of paralogous genes which could generate problems in linkage mapping. The unigene set was analysed to mine perfect SSR sequences using a modified SSRIT perl script [46] using the following criteria: five repeats for dinucleotides, four repeats for tri-, tetra- amd penta-nucleotides, and three repeats for hexa-nucleotides (5,2 - 4,3 - 4,4 - 4,5 - 3,6) for whole analysis and primers design. A pool of 300 non redundant SSR-containing sequences were selected for evaluation of markers polymorphism; they were based on the longer stretch and no more than one SSR per unigene was used. Primers design was carried out using the web interface of BatchPrimer3 (<http://probes.pw.usda.gov/batchprimer>) setting an optimal GC-content of 50%, a max Tm difference of 3°C and variable amplicons size for multiplexed genotyping; all other parameters were set to default values. Newly developed SSR markers were given the prefix CyEM (Cynara Expressed Microsatellite) attached to a number ranging from 1 to 2311.

**Plant materials and genomic DNA isolation**

DNA was extracted from young leaves following Lanteri *et al*. [47]. The primers developed were used to amplify DNA of a set of genotypes including: i) twelve globe artichoke accessions, representative of germplasm grown in Mediterranean Basin [20]; ii) nine cultivated cardoon accessions, representative of the two distinct Spanish and Italian gene pools [21]; iii) seven wild cardoon accessions sampled from both Sicily and Sardinia gene pools defined by Portis *et al.* [27]. The set also included the four parents of three established mapping populations, e.g: two globe artichoke accessions [‘Romanesco C3’ (C3) and ‘Spinoso di Palermo’ (SP)], one cultivated cardoon (‘Altilis 41’) and one wild cardoon (‘Creta 4’); furthermore six F1 individuals from each of the segregating populations (C3 x SP, C3 x Altilis 41 and C3 x Creta 4) were included in the analyses. Full accession details are reported in Table 1.

**Genotyping and diversity analysis**

A total of 300 primer pairs (from CyEM-001 to CyEM-300; Additional file 4: primer pairs designed) were tested for their informativeness on the 28 genotypes reported in Table 1. Amplifications were carried out in 10 µl reactions containing 7 ng template DNA, 1X PCR Buffer (Qiagen Inc., Venlo, Netherlands), 1.0 mM MgCl_2_, 0.5U Taq DNA polymerase (Qiagen), 40 nM 5'-labelled forward primer, 40 nM unlabeled reverse primer and 0.2 mM dNTPs. A touchdown approach was adopted as following: 1 x 94°C\150sec, 9 x 94°C/30sec, 63°C/30sec (-0,7°C/cycle), 72°C/60sec, then 30 x 94°C/30sec, 57°C/30sec, 72°C/60sec, followed by a final extension at 72°C/5min. For weak PCR products 1.5 mM MgCl_2_ and a final annealing temperature of 55°C were adopted. The forward primers were 5’-labelled with FAM, HEX or TAMRA, allowing multiplex amplicons analysis on ABI3730 capillary DNA sequencer (Applied Biosystem Inc., Foster City, CA, USA). Internal ROX-labelled GS500 size standards were included in each capillary. Fragments data were analysed using the software GeneMapper ver. 3.5 (Applied Biosystems).

Genotyping data were scored in Microsoft Excel with alleles sizes in a co-dominant fashion and analysed using the GenAlex Excel package [48]. In order to estimate overall levels of genetic diversity, the following were calculated for all groups of genotypes (globe artichoke, cultivated cardoon and wild cardoon accessions): average number of microsatellite alleles observed per locus (n_o_), effective number of alleles per locus (n_e_), mean observed heterozygosity (H_o_) and polymorphic information content (PIC, estimated as by Anderson *et al*. 1993). A co-phenetic distance matrix for co-dominant markers was generated as described by Smouse and Peakall [49] and used to construct a UPGMA-based dendrogram [50] by means of NTSYS software package v2.10 [51]. Principal co-ordinate analysis (PCO) was carried out via distance matrix with data standardization by GenAlex package, to display the multi-dimensional relationship between accessions.

**Unigenes annotation and categorization**

All the unigenes were aligned against *Arabidopsis* *thaliana* reference proteins database (NCBI) using BlastX algorithm [52]; a threshold of e^-29^ for the E-value was adopted to select significative alignments suitable for unigenes annotation. Microsatellite position (5’-UTR, CDS or 3’-UTR) inside each putative transcript was inferred correlating the data obtained by the SSR-mining to the blatsX alignment, adding an ORF predictor ([https://fungalgenome.concordia.ca](https://fungalgenome.concordia.ca/)) [53] to estimate the real stop codon; data were combined under Microsoft Excel.

The frequencies of aminoacidic repeat stretches in CDS were investigated to assess over-representation of coding triplets. unigenes were splitted in 10 subgroups differing for trinucleotidic motif in CDS. Each subgroup was analysed with ORF-Predictor to show any over-represented aminoacid stretch, since a SSR motif can basically originate 3 types of aminoacidic stretches (e.g. AAG/AGA/GAA can be translated in Lis, Arg or Glu). The analysis was conducted considering only the positive frames (+1, +2, +3), since the sequenced transcripts were originally directionally cloned (Tangs *et al.* 2008, unpublished).

All the unigenes were functionally categorized by GeneOntology on TAIR website ([www.arabidopsis.org](http://www.arabidopsis.org/)); *A. thaliana* accessions resulting from previously described blastX alignment were used as input (intentionally redundat for subject resulting from different queries). Enrichments inside GO hierarchical levels by the mean of different subset of unigenes were extimated through the GoStat2 interface (<http://gostat.wehi.edu.au/cgi-bin/goStat2.pl>) [54], assuming a significance threshold of P < 0,01 on Fisher exact test.

### Authors' contributions

SL and SK planned and supervised the work. DS carried out SSR mining, primer design and amplification; AA and EP selected the set of *C. cardunculus* genotypes in study; DS, AA, EP and CT carried out the analysis of data. All the authors contributed to the final version of the manuscript.

**Acknowledgements**

We are very grateful to Prof. Giovanni Mauromicale and Dr. Rosario Mauro (D.A.C.P.A. Agronomical Sciences, University of Catania), for having provided the *C. cardunculus* parental genotypes and the F_1_ individuals. Globe artichoke EST data was obtained from [**The Compositae Genome Project website**](http://compgenomics.ucdavis.edu/) that was supported by the USDA IFAFS program and NSF Plant Genome Program.

**Figure legends**

**Figure 1**: **Workflow depicting EST assembling, annotation, primer design and screening**.

**Figure 2**: **Assembly output**. (A) Distribution of unigenes length and (B) GC-content.

**Figure 3**: **Information content of analyzed EST-SSR markers** (A) Allelic patterns and level of heterozygosity within each taxa. Number of observed (n_o_), effective (n_e_) and private alleles are reported as bars. Polymorphic information content (PIC) and observed heterozygosity (H_o_) are reported as points. (B) Markers showing segregation on each of the three mapping progeny: “Romanesco C3” x “Spinoso di Palermo” (GxG), “Romanesco C3” x “*altilis*41” (GxC) and “Romanesco C3” x “Creta-4” (GxW).

**Figure 4: Diversity analysis.** (A) UPGMA dendogram of the 28 genotypes based on 1546 EST-SSR alleles. In brackets are reported the globe artichoke, cultivated cardoon and wild cardoon clusters defined in Lanteri *et al.* (2004), Portis *et al*. (2005a) and Portis *et al*. (2005b), respectively. (B) Principal co-ordinates analysis on 46 genotypes via distance matrix with data standardization: individuals from mapping progenies [“Romanesco C3” x “Spinoso di Palermo” (GxG), “Romanesco C3” x “*altilis*41” (GxC) and “Romanesco C3” x “Creta-4” (GxW)] were included; parents are marked by a red circle.

**Figure 5**: **Microsatellites position and motifs**. (A) Distribution within regions of the unigenes. (B)Frequencies of repeat motifs in the unigenes set.

**Figure 6: Distribution of microsatellite classes.** Dinucleotidic and trinucleotidic classes belonging to each unigene regions (5’-UTR, coding sequence, 3-UTR).

**Table 1:** **Genotypes set**.

| ***C. cardunculus* taxa** | **Genotypes** | **Cluster^1^** |
| --- | --- | --- |
| *scolymus*  *altilis*  *sylvestris* | Violet de Provence  AVM 7  Blanco  Pasquaiolo  Pietralcina  Romanesco C3  Green Globe  Sakiz  Spinoso di Palermo  Spinoso violetto di Liguria  Empolese  Violetto di Toscana  Blanco de Peralta  Lleno de España  Rojo de Corella  Valencia  Gigante di Romagna  Bianco Avorio  Gobbo di Nizza  Bianco Pieno Migliorato  Altilis 41  Bronte  Roccella  Palazzolo  Sassari  Oristano  Nuoro  Creta 4 | **A1**  **A1**  **A1**  **A2**  **A2**  **A2**  **A2**  **B1**  **B1**  **B2**  **B2**  **B2**  **A1**  **A1**  **A2**  **A3**  **B1**  **B2**  **B2**  **C**  **-**  **Sicily**  **Sicily**  **Sicily**  **Sardinia**  **Sardinia**  **Sardinia**  **-** |

The 28 *C. cardunculus* accessions assayed for genotypic variation. Respectively 12 globe artichokes, 9 cultivated cardoons and 7 wild cardoons were analysed. **^1^**Globe artichoke, cultivated cardoon and wild cardoon clusters defined in Lanteri *et al.* (2004), Portis *et al*. (2005a) and Portis *et al*. (2005b), respectively.

**Table 2:** **CyEM loci statistics.**

| **Locus** | **Accession N° / unigene name** | **Exp. Size** | **Motif** | **N° of repeats** | **Allele size range** | **n_a_** | **n_e_** | **H_o_** | **PIC** | **GxG** | **GxC** | **GxW** |
| --- | --- | --- | --- | --- | --- | --- | --- | --- | --- | --- | --- | --- |
| **CyEM-001** | GE602982 | 148 | ATAC | 5 | 141-150 | 3 | 2,018 | 0,679 | 0,504 | + | + | + |
| **CyEM-002** | GE604144 | 150 | TTTG | 6 | 139-143 | 2 | 1,733 | 0,464 | 0,423 | + | + | + |
| **CyEM-003** | GE604267 | 144 | CAATGG | 9 | 110-141 | 5 | 3,324 | 0,440 | 0,699 | - | + | - |
| **CyEM-004** | GE604528 | 175 | ATC | 20 | 125-138 | 3 | 1,075 | 0,071 | 0,070 | - | - | - |
| **CyEM-005** | GE605519 | 154 | TC | 9 | 137-170 | 9 | 2,446 | 0,571 | 0,591 | + | - | + |
| **CyEM-006** | GE606838 | 152 | ATC | 13 | 135-162 | 8 | 3,269 | 0,556 | 0,694 | - | - | + |
| **CyEM-007** | GE609770 | 165 | TTC | 17 | 121-165 | 8 | 3,735 | 0,577 | 0,732 | + | + | + |
| **CyEM-008** | GE610358 | 149 | CAT | 19 | 106-156 | 9 | 3,347 | 0,654 | 0,701 | + | + | + |
| **CyEM-009** | GE610418 | 143 | ATAC | 6 | 118-146 | 10 | 6,693 | 0,500 | 0,851 | + | + | + |
| **CyEM-010** | GE612656 | 180 | TGTA | 7 | 152-194 | 12 | 7,259 | 0,643 | 0,862 | + | + | + |
| **CyEM-011** | GE613193 | 209 | TTGGTT | 10 | 170-214 | 5 | 3,057 | 0,393 | 0,673 | - | + | + |
| **CyEM-012** | GE578689 | 153 | GAA | 8 | 146-169 | 8 | 4,036 | 0,538 | 0,752 | + | + | + |
| **CyEM-013** | GE588213 | 149 | GAT | 10 | 124-151 | 8 | 3,416 | 0,429 | 0,707 | + | + | + |
| **CyEM-014** | GE595596 | 170 | ATG | 11 | 153-172 | 7 | 4,308 | 0,643 | 0,768 | + | + | + |
| **CyEM-015** | CL2994Contig1 | 92 | GTTT | 5 | 84-97 | 4 | 2,592 | 0,750 | 0,614 | + | + | + |
| **CyEM-016** | GE587666 | 127 | TATG | 5 | 117-129 | 4 | 2,922 | 0,481 | 0,658 | + | + | + |
| **CyEM-017** | CL469Contig1 | 187 | TTGGT | 6 | 173-205 | 7 | 3,588 | 0,429 | 0,721 | - | - | + |
| **CyEM-018** | CL6299Contig1 | 156 | GGTCT | 6 | 139-156 | 2 | 1,813 | 0,321 | 0,448 | - | - | - |
| **CyEM-019*** | CL2425Contig1 | 179 | TGGTA | 6 | - | \ | \ | \ | \ | + | + | + |
| **CyEM-020** | GE612053 | 107 | TCATCT | 6 | 67-104 | 7 | 3,724 | 0,321 | 0,732 | + | + | + |
| **CyEM-021** | CL8Contig1 | 231 | CGC | 5 | 234-234 | 1 | 1,000 | 0,000 | 0,000 | - | - | - |
| **CyEM-022** | CL167Contig1 | 222 | ATG | 5 | 224-224 | 1 | 1,000 | 0,000 | 0,000 | - | - | - |
| **CyEM-023** | CL290Contig1 | 234 | CT | 5 | 214-233 | 4 | 1,675 | 0,429 | 0,403 | - | + | - |
| **CyEM-024** | CL432Contig1 | 125 | ATC | 7 | 113-126 | 4 | 3,655 | 0,464 | 0,726 | + | + | + |
| **CyEM-025** | CL489Contig1 | 229 | CTA | 6 | 229-239 | 3 | 1,878 | 0,321 | 0,467 | + | + | + |
| **CyEM-027** | CL768Contig1 | 228 | ACC | 6 | 224-227 | 2 | 1,036 | 0,036 | 0,035 | - | - | - |
| **CyEM-028** | CL1480Contig1 | 228 | CGATTA | 7 | 530-544 | 4 | 1,845 | 0,095 | 0,458 | + | - | + |
| **CyEM-029** | CL2522Contig1 | 219 | CTTC | 7 | 202-223 | 5 | 3,315 | 0,607 | 0,698 | + | + | + |
| **CyEM-030** | CL2739Contig1 | 240 | TC | 19 | 212-245 | 11 | 7,245 | 0,667 | 0,862 | - | + | + |
| **CyEM-031** | CL2833Contig1 | 223 | CT | 8 | 117-226 | 7 | 3,682 | 0,333 | 0,728 | + | - | + |
| **CyEM-032** | CL5674Contig1 | 229 | CAT | 8 | 214-236 | 5 | 3,294 | 0,500 | 0,696 | + | + | + |
| **CyEM-033** | CL6305Contig1 | 227 | CTT | 12 | 205-231 | 8 | 3,213 | 0,571 | 0,689 | + | - | + |
| **CyEM-034** | CL6392Contig1 | 212 | CT | 17 | 185-224 | 9 | 6,078 | 0,750 | 0,835 | + | + | + |
| **CyEM-035** | CL136Contig1 | 231 | GGTTA | 5 | 212-239 | 6 | 3,435 | 0,739 | 0,709 | + | + | + |
| **CyEM-036** | CL840Contig1 | 231 | GAATT | 5 | 222-237 | 4 | 2,379 | 0,500 | 0,580 | + | + | + |
| **CyEM-037** | CL1651Contig1 | 220 | CT | 16 | 191-225 | 12 | 6,759 | 0,679 | 0,852 | + | + | + |
| **CyEM-038** | CL3137Contig1 | 220 | AAGTG | 5 | 216-226 | 3 | 2,316 | 0,536 | 0,568 | + | + | + |
| **CyEM-042** | CL4773Contig1 | 168 | AG | 14 | 148-176 | 12 | 6,438 | 0,577 | 0,845 | + | + | + |
| **CyEM-043** | CL5064Contig1 | 292 | ACA | 10 | 272-297 | 7 | 4,021 | 0,500 | 0,751 | + | + | + |
| **CyEM-045** | CL5134Contig1 | 301 | CAATC | 5 | 290-299 | 3 | 1,651 | 0,280 | 0,394 | + | + | + |
| **CyEM-046** | CL5445Contig1 | 278 | CTTTGC | 5 | 273-278 | 2 | 1,415 | 0,071 | 0,293 | - | - | + |
| **CyEM-047** | CL6123Contig1 | 286 | CATCTT | 5 | 274-303 | 5 | 3,226 | 0,571 | 0,690 | + | + | + |
| **CyEM-048** | CL703Contig1 | 292 | CAATCC | 5 | 270-294 | 6 | 3,378 | 0,560 | 0,704 | + | + | + |
| **CyEM-049** | CL1527Contig1 | 305 | CAGAAG | 6 | 284-307 | 5 | 3,246 | 0,393 | 0,692 | + | - | - |
| **CyEM-050** | CL1584Contig1 | 311 | TTGGT | 5 | 269-315 | 8 | 1,617 | 0,240 | 0,382 | - | - | + |
| **CyEM-051** | CL1735Contig1 | 162 | TGGCAA | 5 | 129-167 | 5 | 1,551 | 0,074 | 0,355 | - | - | - |
| **CyEM-052** | CL1878Contig1 | 301 | CT | 15 | 277-303 | 11 | 6,788 | 0,964 | 0,853 | + | + | + |
| **CyEM-053** | CL2037Contig1 | 327 | CT | 20 | 297-330 | 13 | 8,667 | 0,885 | 0,885 | + | + | + |
| **CyEM-054** | CL4038Contig1 | 307 | ATGTGG | 6 | 268-305 | 11 | 9,191 | 0,600 | 0,891 | + | + | + |
| **CyEM-055** | CL4185Contig1 | 297 | CAACAG | 7 | 271-293 | 5 | 3,995 | 0,630 | 0,750 | + | + | + |
| **CyEM-056** | CL5289Contig1 | 301 | GA | 21 | 283-319 | 9 | 4,404 | 0,385 | 0,773 | + | + | + |
| **CyEM-057** | CL6231Contig1 | 301 | AT | 6 | 300-323 | 6 | 2,620 | 0,308 | 0,618 | + | + | + |
| **CyEM-058** | CL815Contig1 | 299 | CA | 7 | 291-298 | 2 | 1,642 | 0,000 | 0,391 | - | + | + |
| **CyEM-059** | CL1157Contig1 | 289 | GGT | 9 | 280-297 | 7 | 2,116 | 0,429 | 0,527 | + | + | + |
| **CyEM-060** | CL1449Contig1 | 306 | GATTC | 5 | 294-307 | 4 | 2,605 | 0,429 | 0,616 | + | + | + |
| **CyEM-063** | GE590526 | 375 | GATGG | 5 | 432-457 | 8 | 3,673 | 0,481 | 0,728 | + | + | + |
| **CyEM-064** | GE590983 | 122 | CT | 15 | 96-126 | 12 | 8,145 | 0,741 | 0,877 | + | + | + |
| **CyEM-066** | GE591829 | 368 | GAT | 15 | 372-405 | 10 | 6,231 | 0,704 | 0,840 | + | + | + |
| **CyEM-069** | GE594774 | 386 | AGGA | 5 | 370-392 | 6 | 3,556 | 0,519 | 0,719 | - | + | + |
| **CyEM-070** | GE594818 | 272 | TGCA | 5 | 309-380 | 5 | 3,769 | 0,393 | 0,735 | + | + | + |
| **CyEM-071** | GE595888 | 376 | GTTTG | 5 | 438-468 | 6 | 2,713 | 0,357 | 0,631 | - | - | - |
| **CyEM-072** | GE595959 | 376 | AAGCA | 5 | 367-386 | 4 | 3,308 | 0,536 | 0,698 | + | + | + |
| **CyEM-073** | GE596794 | 376 | AGCC | 6 | 457-465 | 4 | 2,088 | 0,286 | 0,521 | + | + | - |
| **CyEM-075** | GE597515 | 390 | TC | 16 | 363-393 | 7 | 3,458 | 0,462 | 0,711 | - | + | + |
| **CyEM-076** | GE597588 | 378 | AACCA | 14 | 436-449 | 6 | 3,627 | 0,556 | 0,724 | + | + | + |
| **CyEM-077** | GE598177 | 378 | CCAT | 6 | 370-380 | 5 | 3,492 | 0,429 | 0,714 | + | + | + |
| **CyEM-079** | GE601502 | 375 | AATG | 6 | 463-488 | 8 | 5,333 | 0,625 | 0,813 | + | + | + |
| **CyEM-080** | GE602408 | 382 | TTCACG | 14 | 652-694 | 4 | 3,273 | 0,714 | 0,695 | + | + | + |
| **CyEM-083** | CL5605Contig1 | 465 | AG | 13 | 448-475 | 7 | 3,689 | 0,643 | 0,729 | + | - | - |
| **CyEM-084** | CL5717Contig1 | 128 | AATCA | 5 | 108-123 | 3 | 2,018 | 0,429 | 0,504 | + | + | + |
| **CyEM-086** | GE577139 | 430 | ATGTAA | 6 | 410-460 | 6 | 3,447 | 0,389 | 0,710 | + | + | + |
| **CyEM-087** | GE578205 | 450 | CCAAC | 5 | 443-457 | 5 | 1,488 | 0,125 | 0,328 | - | - | - |
| **CyEM-088** | GE578232 | 451 | GAGGAA | 7 | 436-459 | 5 | 2,045 | 0,222 | 0,511 | - | - | + |
| **CyEM-090** | GE580735 | 201 | ATAC | 6 | 190-218 | 5 | 1,615 | 0,222 | 0,381 | - | + | - |
| **CyEM-091** | GE581152 | 452 | GGTAT | 5 | 657-669 | 4 | 2,493 | 0,464 | 0,599 | + | + | + |
| **CyEM-092** | GE581504 | 106 | TTGC | 7 | 83-104 | 6 | 4,683 | 0,750 | 0,786 | - | - | - |
| **CyEM-093** | GE581834 | 435 | GA | 18 | 410-453 | 12 | 7,362 | 0,857 | 0,864 | + | + | + |
| **CyEM-094** | GE581842 | 450 | TCA | 14 | 417-454 | 5 | 2,068 | 0,333 | 0,516 | + | + | + |
| **CyEM-096** | GE586326 | 451 | CTCTAT | 6 | 426-465 | 9 | 3,144 | 0,346 | 0,682 | + | - | - |
| **CyEM-097** | GE587846 | 446 | GT | 12 | 437-449 | 5 | 3,415 | 0,556 | 0,707 | + | + | + |
| **CyEM-098** | GE588210 | 262 | AAGAG | 5 | 620-650 | 4 | 3,068 | 0,357 | 0,674 | - | - | - |
| **CyEM-099** | GE588482 | 448 | AAGTG | 5 | 536-547 | 3 | 2,594 | 0,593 | 0,615 | + | + | + |
| **CyEM-100** | GE589916 | 434 | AT | 11 | 522-554 | 10 | 6,857 | 0,375 | 0,854 | - | - | + |
| **CyEM-102** | GE590134 | 100 | ACC | 7 | 87-105 | 5 | 1,871 | 0,250 | 0,466 | - | + | + |
| **CyEM-103** | GE592369 | 100 | AGC | 7 | 93-105 | 4 | 1,821 | 0,571 | 0,451 | + | - | + |
| **CyEM-104** | GE595980 | 100 | CAG | 7 | 94-117 | 8 | 4,226 | 0,679 | 0,763 | + | + | + |
| **CyEM-105** | GE588534 | 101 | AAG | 7 | 85-107 | 6 | 3,355 | 0,654 | 0,702 | + | + | + |
| **CyEM-106** | GE588636 | 101 | CAG | 7 | 84-99 | 6 | 3,908 | 0,692 | 0,744 | + | + | + |
| **CyEM-107** | GE591921 | 101 | GAA | 7 | 91-115 | 6 | 3,391 | 0,704 | 0,705 | + | + | + |
| **CyEM-108** | GE590638 | 102 | ACA | 7 | 293-356 | 12 | 5,481 | 0,778 | 0,818 | + | + | + |
| **CyEM-109** | GE586147 | 103 | GGA | 7 | 50-102 | 7 | 3,980 | 0,357 | 0,749 | - | - | + |
| **CyEM-110** | GE586350 | 103 | GTT | 7 | 98-105 | 3 | 1,618 | 0,393 | 0,382 | - | + | - |
| **CyEM-111** | GE587414 | 165 | CGG | 7 | 105-124 | 6 | 4,467 | 0,464 | 0,776 | + | + | + |
| **CyEM-112** | GE593991 | 104 | TCA | 7 | 99-117 | 7 | 4,519 | 0,679 | 0,779 | + | + | + |
| **CyEM-113** | GE584535 | 107 | CAC | 7 | 97-102 | 3 | 1,332 | 0,286 | 0,249 | + | + | + |
| **CyEM-115** | GE582326 | 109 | CTG | 7 | 94-120 | 6 | 2,190 | 0,179 | 0,543 | - | - | - |
| **CyEM-117** | GE596127 | 110 | GAT | 7 | 103-109 | 3 | 1,742 | 0,429 | 0,426 | + | + | + |
| **CyEM-118** | GE597580 | 111 | GCT | 7 | 96-117 | 6 | 3,197 | 0,593 | 0,687 | + | + | + |
| **CyEM-120** | GE597566 | 113 | ATT | 7 | 99-120 | 6 | 3,350 | 0,464 | 0,702 | + | + | + |
| **CyEM-121** | GE590328 | 114 | ACA | 7 | 265-287 | 7 | 3,574 | 0,519 | 0,720 | + | + | + |
| **CyEM-122** | GE583054 | 115 | CTG | 7 | 118-133 | 5 | 2,149 | 0,385 | 0,535 | - | + | + |
| **CyEM-123** | GE592105 | 115 | GTG | 7 | 107-120 | 3 | 1,640 | 0,500 | 0,390 | + | + | + |
| **CyEM-124** | GE597437 | 117 | GT | 12 | 112-126 | 8 | 4,598 | 0,571 | 0,783 | + | + | + |
| **CyEM-126** | GE601086 | 119 | AGC | 8 | 111-158 | 7 | 2,834 | 0,731 | 0,647 | - | + | + |
| **CyEM-127** | GE586328 | 110 | CCA | 8 | 99-116 | 7 | 3,960 | 0,857 | 0,747 | + | + | + |
| **CyEM-128** | CL4629Contig1 | 120 | AG | 12 | 103-131 | 10 | 2,830 | 0,500 | 0,647 | + | + | + |
| **CyEM-129** | GE594087 | 123 | AGT | 8 | 105-124 | 5 | 2,649 | 0,250 | 0,622 | - | - | - |
| **CyEM-130** | GE610344 | 123 | GAT | 8 | 112-131 | 6 | 3,168 | 0,393 | 0,684 | + | + | + |
| **CyEM-131** | GE580155 | 258 | TC | 12 | 260-296 | 9 | 4,989 | 0,538 | 0,800 | - | - | - |
| **CyEM-132** | GE589900 | 126 | GTG | 8 | 112-127 | 5 | 2,851 | 0,286 | 0,649 | - | - | + |
| **CyEM-133** | GE582083 | 128 | CAT | 8 | 121-155 | 10 | 3,806 | 0,464 | 0,737 | - | + | + |
| **CyEM-134** | GE587520 | 128 | TGA | 8 | 123-129 | 3 | 2,402 | 0,407 | 0,584 | + | + | + |
| **CyEM-135** | GE580164 | 129 | TC | 12 | 125-151 | 10 | 6,426 | 0,893 | 0,844 | + | + | + |
| **CyEM-136** | GE599224 | 129 | GA | 12 | 118-145 | 10 | 5,502 | 0,679 | 0,818 | + | + | + |
| **CyEM-138** | CL2919Contig1 | 130 | TC | 12 | 112-153 | 13 | 6,788 | 0,714 | 0,853 | + | + | + |
| **CyEM-139** | CL5080Contig1 | 130 | CAA | 8 | 122-139 | 6 | 2,835 | 0,500 | 0,647 | + | - | + |
| **CyEM-141** | GE588755 | 133 | ATC | 8 | 119-134 | 5 | 3,019 | 0,593 | 0,669 | + | + | + |
| **CyEM-142** | GE581587 | 134 | CAT | 8 | 119-140 | 5 | 2,246 | 0,231 | 0,555 | - | - | - |
| **CyEM-143** | GE577330 | 135 | AG | 12 | 125-145 | 8 | 3,540 | 0,357 | 0,717 | - | - | + |
| **CyEM-144** | GE602230 | 136 | TGA | 8 | 128-158 | 6 | 3,853 | 0,679 | 0,740 | + | + | + |
| **CyEM-145** | GE594958 | 139 | GAT | 8 | 133-181 | 8 | 3,807 | 0,519 | 0,737 | + | + | + |
| **CyEM-146** | CL4926Contig1 | 164 | CCA | 6 | 226-241 | 7 | 2,259 | 0,143 | 0,557 | + | + | - |
| **CyEM-147** | CL2920Contig1 | 165 | CTC | 7 | 140-164 | 7 | 2,864 | 0,286 | 0,651 | + | + | + |
| **CyEM-148** | GE608083 | 167 | GAT | 6 | 161-179 | 5 | 2,420 | 0,250 | 0,587 | - | + | + |
| **CyEM-149** | GE605451 | 168 | CA | 10 | 115-124 | 3 | 2,378 | 0,222 | 0,580 | - | - | - |
| **CyEM-150** | GE588087 | 169 | AG | 10 | 138-171 | 9 | 4,681 | 0,286 | 0,786 | + | + | + |
| **CyEM-151** | CL1781Contig1 | 169 | CAC | 7 | 150-169 | 6 | 3,136 | 0,429 | 0,681 | + | - | + |
| **CyEM-152** | GE579243 | 170 | GA | 10 | 165-187 | 8 | 4,976 | 0,407 | 0,799 | + | + | + |
| **CyEM-153** | GE603802 | 171 | AAC | 6 | 165-183 | 6 | 2,450 | 0,393 | 0,592 | - | + | + |
| **CyEM-154** | GE581295 | 102 | TC | 10 | 94-106 | 7 | 1,653 | 0,423 | 0,395 | + | - | + |
| **CyEM-155** | GE608129 | 173 | CAG | 6 | 165-174 | 4 | 2,877 | 0,571 | 0,652 | + | + | + |
| **CyEM-156** | GE596512 | 174 | TC | 10 | 171-185 | 6 | 4,094 | 0,464 | 0,756 | + | + | + |
| **CyEM-157** | CL5016Contig1 | 174 | TGA | 6 | 154-197 | 8 | 6,698 | 0,458 | 0,851 | + | + | + |
| **CyEM-158** | GE599088 | 200 | TCA | 6 | 193-199 | 2 | 1,899 | 0,308 | 0,473 | + | + | + |
| **CyEM-159** | GE612769 | 176 | TC | 10 | 167-187 | 9 | 3,260 | 0,571 | 0,693 | + | - | + |
| **CyEM-160** | CL3274Contig1 | 176 | CTT | 7 | 163-185 | 6 | 1,589 | 0,286 | 0,371 | - | - | - |
| **CyEM-162** | CL1575Contig1 | 258 | TAG | 7 | 253-261 | 5 | 2,914 | 0,500 | 0,657 | + | + | + |
| **CyEM-163** | GE595958 | 179 | AC | 10 | 174-190 | 8 | 4,442 | 0,607 | 0,775 | + | + | + |
| **CyEM-164** | GE583509 | 182 | AC | 10 | 380-401 | 5 | 3,406 | 0,440 | 0,706 | + | + | + |
| **CyEM-165** | GE581808 | 188 | TC | 10 | 182-194 | 4 | 3,004 | 0,393 | 0,667 | - | - | - |
| **CyEM-166*** | GE605263 | 189 | CT | 10 | - | \ |  | \ | \ | + | + | + |
| **CyEM-167** | CL1848Contig1 | 189 | AG | 11 | 173-212 | 13 | 7,095 | 0,643 | 0,859 | + | + | + |
| **CyEM-169** | GE610017 | 190 | CTT | 7 | 176-191 | 4 | 1,958 | 0,393 | 0,489 | + | + | + |
| **CyEM-170** | GE598301 | 192 | TGG | 7 | 186-198 | 4 | 1,292 | 0,179 | 0,226 | - | - | + |
| **CyEM-171** | GE610233 | 194 | TCA | 7 | 196-252 | 8 | 3,136 | 0,429 | 0,681 | - | - | + |
| **CyEM-172** | CL5891Contig1 | 195 | CT | 11 | 394-432 | 13 | 4,532 | 0,393 | 0,779 | + | + | + |
| **CyEM-173** | GE607339 | 196 | GAC | 7 | 187-204 | 6 | 2,777 | 0,593 | 0,640 | + | + | + |
| **CyEM-174** | GE609927 | 196 | CCA | 7 | 109-209 | 7 | 3,503 | 0,538 | 0,714 | + | + | + |
| **CyEM-175** | CL6045Contig1 | 197 | CT | 11 | 190-211 | 6 | 5,058 | 0,643 | 0,802 | + | + | + |
| **CyEM-176** | GE604158 | 198 | ACA | 7 | 191-203 | 4 | 3,588 | 0,393 | 0,721 | + | + | + |
| **CyEM-178** | GE612882 | 200 | CAG | 7 | 188-202 | 5 | 2,883 | 0,385 | 0,653 | + | + | + |
| **CyEM-179** | GE601991 | 201 | ATG | 7 | 194-203 | 3 | 1,338 | 0,000 | 0,253 | - | - | - |
| **CyEM-180** | CL431Contig1 | 201 | CT | 11 | 194-202 | 5 | 2,920 | 0,536 | 0,658 | - | - | + |
| **CyEM-181** | GE577085 | 108 | GT | 11 | 96-111 | 8 | 4,599 | 0,593 | 0,783 | + | + | + |
| **CyEM-182** | GE607197 | 202 | TCA | 7 | 192-225 | 9 | 6,453 | 0,393 | 0,845 | + | - | + |
| **CyEM-183** | GE597664 | 98 | CAA | 7 | 91-103 | 5 | 3,174 | 0,786 | 0,685 | + | + | + |
| **CyEM-185** | GE610261 | 208 | GAA | 7 | 183-296 | 12 | 6,258 | 0,741 | 0,840 | + | + | + |
| **CyEM-186** | GE607652 | 209 | CTC | 7 | 198-216 | 7 | 4,695 | 0,536 | 0,787 | - | - | - |
| **CyEM-187** | GE602677 | 210 | AGC | 7 | 255-261 | 3 | 1,075 | 0,071 | 0,070 | - | - | - |
| **CyEM-188** | GE613233 | 210 | GCA | 7 | 194-225 | 10 | 6,288 | 0,538 | 0,841 | + | + | + |
| **CyEM-189** | GE605002 | 215 | ATC | 7 | 211-220 | 4 | 2,877 | 0,571 | 0,652 | + | + | + |
| **CyEM-190** | CL1046Contig1 | 242 | CAG | 7 | 235-250 | 6 | 3,530 | 0,630 | 0,717 | + | + | + |
| **CyEM-193** | CL1609Contig2 | 246 | ATC | 7 | 254-268 | 6 | 2,256 | 0,571 | 0,557 | + | + | + |
| **CyEM-195** | CL6448Contig1 | 249 | ACA | 7 | 397-420 | 7 | 3,778 | 0,357 | 0,735 | + | + | + |
| **CyEM-196** | GE580410 | 250 | CAT | 7 | 238-267 | 8 | 3,919 | 0,231 | 0,745 | + | + | + |
| **CyEM-197** | CL3269Contig1 | 250 | TGA | 7 | 245-274 | 7 | 3,496 | 0,370 | 0,714 | - | + | - |
| **CyEM-199** | CL3496Contig1 | 251 | ATC | 7 | 246-260 | 8 | 6,426 | 0,679 | 0,844 | + | + | + |
| **CyEM-200** | CL4126Contig1 | 237 | CAT | 7 | 217-259 | 6 | 3,471 | 0,519 | 0,712 | + | + | + |
| **CyEM-201** | CL6303Contig1 | 252 | GAA | 7 | 225-251 | 6 | 2,521 | 0,214 | 0,603 | + | + | - |
| **CyEM-202** | CL2754Contig1 | 105 | CAG | 7 | 92-106 | 5 | 4,084 | 0,630 | 0,755 | + | + | + |
| **CyEM-203** | CL2776Contig1 | 253 | ACC | 7 | 325-330 | 3 | 2,074 | 0,333 | 0,518 | - | - | + |
| **CyEM-204** | CL5986Contig1 | 253 | TCT | 7 | 242-263 | 7 | 3,769 | 0,321 | 0,735 | - | + | - |
| **CyEM-205** | CL3033Contig1 | 237 | CAC | 7 | 230-239 | 4 | 3,073 | 0,462 | 0,675 | + | + | + |
| **CyEM-207** | CL4470Contig1 | 257 | CAC | 7 | 240-268 | 10 | 6,231 | 0,741 | 0,840 | + | + | + |
| **CyEM-208** | CL5699Contig1 | 257 | AAG | 7 | 254-256 | 2 | 1,080 | 0,000 | 0,074 | - | - | - |
| **CyEM-209** | CL1652Contig1 | 188 | ATC | 7 | 180-194 | 5 | 1,576 | 0,143 | 0,365 | + | + | + |
| **CyEM-210** | GE611460 | 260 | TGA | 8 | 253-267 | 6 | 3,446 | 0,478 | 0,710 | + | + | + |
| **CyEM-211** | CL6394Contig1 | 120 | GCA | 7 | 114-123 | 4 | 2,116 | 0,370 | 0,527 | + | + | + |
| **CyEM-212** | CL2349Contig1 | 262 | ACC | 7 | 262-268 | 3 | 1,124 | 0,038 | 0,110 | - | - | - |
| **CyEM-213** | GE603351 | 263 | TC | 13 | 235-283 | 12 | 7,801 | 0,821 | 0,872 | + | - | + |
| **CyEM-214** | CL1016Contig1 | 263 | CAT | 9 | 253-293 | 12 | 5,045 | 0,731 | 0,802 | + | + | + |
| **CyEM-215** | CL6059Contig1 | 263 | CAC | 9 | 258-274 | 4 | 2,379 | 0,214 | 0,580 | - | - | + |
| **CyEM-216** | GE578065 | 264 | CAT | 9 | 229-265 | 9 | 3,707 | 0,321 | 0,730 | - | - | + |
| **CyEM-218** | GE611429 | 265 | TC | 13 | 350-370 | 9 | 5,580 | 0,536 | 0,821 | + | + | + |
| **CyEM-219** | GE611316 | 267 | CAC | 8 | 256-278 | 6 | 2,925 | 0,464 | 0,658 | + | + | - |
| **CyEM-220** | CL4549Contig1 | 274 | CAT | 9 | 261-276 | 6 | 4,326 | 0,704 | 0,769 | + | + | + |
| **CyEM-221** | GE611385 | 275 | TG | 12 | 254-282 | 7 | 5,042 | 0,679 | 0,802 | + | + | + |
| **CyEM-223** | CL5961Contig1 | 276 | CAC | 9 | 263-275 | 5 | 2,296 | 0,500 | 0,564 | + | + | + |
| **CyEM-225** | GE580984 | 278 | GAA | 9 | 274-286 | 5 | 3,081 | 0,571 | 0,675 | + | + | + |
| **CyEM-226** | GE611110 | 280 | AGA | 8 | 517-529 | 4 | 3,817 | 0,370 | 0,738 | + | + | + |
| **CyEM-227** | CL5817Contig1 | 280 | AAG | 9 | 241-281 | 6 | 3,142 | 0,667 | 0,682 | + | + | - |
| **CyEM-228** | CL4460Contig1 | 283 | GAT | 9 | 348-357 | 4 | 1,566 | 0,321 | 0,362 | + | - | + |
| **CyEM-229** | GE577281 | 285 | CT | 13 | 270-294 | 10 | 6,222 | 0,786 | 0,839 | + | + | + |
| **CyEM-230** | CL1174Contig1 | 285 | CCA | 9 | 271-293 | 6 | 3,159 | 0,462 | 0,683 | + | + | + |
| **CyEM-231*** | CL548Contig1 | 286 | AGA | 9 | - | \ | \ | \ | \ | + | + | + |
| **CyEM-232** | CL4621Contig1 | 287 | CAG | 9 | 273-287 | 6 | 4,915 | 0,679 | 0,797 | + | + | + |
| **CyEM-233** | GE583211 | 288 | AC | 13 | 444-471 | 11 | 3,672 | 0,679 | 0,728 | + | + | + |
| **CyEM-234*** | GE602543 | 310 | TC | 11 | - | \ | \ | \ | \ | + | + | + |
| **CyEM-236** | CL923Contig1 | 315 | CTT | 8 | 401-433 | 6 | 3,729 | 0,667 | 0,732 | + | + | + |
| **CyEM-237** | GE589921 | 316 | CT | 11 | 310-331 | 10 | 6,202 | 0,885 | 0,839 | + | + | + |
| **CyEM-238** | CL1788Contig1 | 321 | TC | 12 | 304-334 | 9 | 2,904 | 0,296 | 0,656 | - | - | - |
| **CyEM-240** | CL2307Contig1 | 324 | AGC | 8 | 303-333 | 8 | 5,063 | 0,630 | 0,802 | + | + | + |
| **CyEM-241** | CL2526Contig1 | 325 | CT | 12 | 311-325 | 3 | 2,263 | 0,179 | 0,558 | - | - | - |
| **CyEM-243** | CL5805Contig1 | 325 | CT | 12 | 309-338 | 10 | 4,472 | 0,667 | 0,776 | + | + | + |
| **CyEM-244** | GE609380 | 326 | TC | 11 | 318-337 | 9 | 5,080 | 0,519 | 0,803 | + | + | + |
| **CyEM-246** | GE604318 | 327 | TG | 11 | 320-354 | 8 | 3,117 | 0,520 | 0,679 | + | + | + |
| **CyEM-247** | CL2951Contig1 | 327 | CTG | 8 | 316-339 | 7 | 3,815 | 0,500 | 0,738 | + | + | + |
| **CyEM-248** | GE581850 | 330 | AG | 11 | 325-331 | 4 | 2,379 | 0,714 | 0,580 | + | + | + |
| **CyEM-250** | CL3943Contig1 | 331 | AG | 10 | 321-334 | 5 | 3,516 | 0,321 | 0,716 | + | + | + |
| **CyEM-253** | CL3338Contig1 | 142 | CT | 15 | 125-159 | 9 | 4,653 | 0,571 | 0,785 | + | - | + |
| **CyEM-254** | CL3757Contig1 | 125 | TC | 12 | 108-141 | 15 | 7,682 | 0,654 | 0,870 | + | + | + |
| **CyEM-256** | CL6387Contig1 | 338 | TCA | 8 | 323-350 | 7 | 4,148 | 0,464 | 0,759 | - | + | - |
| **CyEM-259** | CL5381Contig1 | 197 | CA | 15 | 176-220 | 12 | 6,826 | 0,696 | 0,853 | + | + | + |
| **CyEM-260** | CL2855Contig1 | 151 | CCA | 10 | 131-151 | 7 | 4,780 | 0,630 | 0,791 | + | + | + |
| **CyEM-261** | GE613227 | 185 | GGT | 9 | 168-202 | 9 | 5,765 | 0,643 | 0,827 | + | + | + |
| **CyEM-264** | CL3958Contig1 | 350 | GAT | 10 | 611-625 | 5 | 3,636 | 0,593 | 0,725 | + | + | + |
| **CyEM-266** | GE598991 | 352 | AG | 14 | 343-359 | 8 | 5,985 | 0,571 | 0,833 | + | + | + |
| **CyEM-272** | GE599540 | 356 | TAC | 9 | 348-357 | 4 | 3,333 | 0,600 | 0,700 | + | + | + |
| **CyEM-273*** | GE599578 | 356 | AAC | 9 | - | \ |  | \ | \ | + | + | + |
| **CyEM-277** | CL2561Contig1 | 138 | TC | 14 | 116-157 | 13 | 5,911 | 0,783 | 0,831 | + | + | + |
| **CyEM-278** | GE579023 | 391 | CAT | 10 | 376-399 | 5 | 3,057 | 0,259 | 0,673 | + | - | + |
| **CyEM-279** | CL4781Contig1 | 106 | TC | 17 | 90-105 | 7 | 5,507 | 0,720 | 0,818 | + | + | + |
| **CyEM-280** | GE591354 | 394 | ATG | 11 | 375-432 | 12 | 7,010 | 0,852 | 0,857 | + | + | + |
| **CyEM-281** | GE610121 | 395 | TC | 15 | 372-412 | 9 | 5,302 | 0,778 | 0,811 | + | - | + |
| **CyEM-282** | GE604802 | 399 | GAT | 11 | 380-401 | 5 | 4,085 | 0,423 | 0,755 | + | + | + |
| **CyEM-284** | CL2318Contig1 | 111 | AG | 17 | 88-114 | 9 | 5,209 | 0,679 | 0,808 | + | + | + |
| **CyEM-285** | CL4633Contig1 | 109 | CT | 16 | 91-129 | 13 | 6,284 | 0,667 | 0,841 | + | - | + |
| **CyEM-286** | GE602088 | 405 | TC | 17 | 386-410 | 9 | 5,074 | 0,679 | 0,803 | + | + | + |
| **CyEM-288** | GE595961 | 410 | ATG | 10 | 388-425 | 7 | 3,072 | 0,542 | 0,674 | + | + | + |
| **CyEM-289** | GE580749 | 411 | CTT | 12 | 461-501 | 4 | 1,247 | 0,214 | 0,198 | + | + | + |
| **CyEM-290** | GE583378 | 160 | TC | 15 | 151-174 | 9 | 5,383 | 0,500 | 0,814 | + | - | - |
| **CyEM-291** | CL1901Contig1 | 147 | TG | 18 | 120-164 | 9 | 3,540 | 0,607 | 0,717 | + | + | + |
| **CyEM-293** | CL3287Contig1 | 413 | TC | 20 | 384-410 | 9 | 4,717 | 0,440 | 0,788 | + | + | + |
| **CyEM-294** | GE593962 | 231 | GAT | 11 | 216-232 | 7 | 4,556 | 0,407 | 0,781 | + | + | + |
| **CyEM-295** | CL2047Contig1 | 414 | AG | 16 | 480-517 | 9 | 3,282 | 0,500 | 0,695 | + | + | + |
| **CyEM-296** | GE602341 | 415 | GAT | 11 | 397-428 | 8 | 5,629 | 0,593 | 0,822 | + | + | + |
| **CyEM-299** | CL6551Contig1 | 424 | CAC | 12 | 469-520 | 9 | 2,254 | 0,333 | 0,556 | + | - | + |
| **CyEM-300** | GE610516 | 425 | TC | 15 | 405-432 | 7 | 3,885 | 0,308 | 0,743 | + | + | + |
| **Average** |  |  |  |  |  | 6,6 | 3,677 | 0,484 | 0,660 |  |  |  |
| **s.e.** |  |  |  |  |  | 0,2 | 0,107 | 0,013 | 0,012 |  |  |  |

Main information reported: locus name, unigene (for contigs-derived loci)/Accession number (for singletons-derived loci), expected size, perfect microsatellite motif, number of repeats, observed alleles range, number of observed alleles (n_o_), effective alleles (n_e_), observed heterozygosity (H_o_), polymorphic information content (PIC) and mapping utility for the three progenies [“Romanesco C3” x “Spinoso di Palermo” (GxG), “Romanesco C3” x “*altilis*41” (GxC) and “Romanesco C3” x “Creta-4” (GxW)]. Additional information are available in the *electronic supplementary material*. *Data showing a multi-locus amplification were excluded from the analysis.

**Table 3: Functional enrichments.**

|  |  |  |  |  | **CDS** | | | | | |  | **5'-UTR** | | | |
| --- | --- | --- | --- | --- | --- | --- | --- | --- | --- | --- | --- | --- | --- | --- | --- |
|  | **ID** | **Name** | **unigenes** | **All SSRs** | **All SSRs** | **TRI** | **DI** | **AAG_CTT** | **ATC_GAT** | **AG_CT** |  | **All SSRs** | **DI** | **TRI** | **AG_CT** |
| Biological process | GO:0006139 | nucleobase, nucleoside, nucleotide and nucleic acid metabolic process | 7,33% | 9,58% | 10,90% | 10,88% | - | - | - | - |  | - | - | - | - |
|  | GO:0019219 | regulation of nucleobase, nucleoside, nucleotide and nucleic acid metabolic process | 3,30% | 5,85% | 7,56% | 8,21% | - | - | 9,09% | - |  | - | - | - | 7,86% |
|  | GO:0006350 | transcription | 3,56% | 5,85% | 7,70% | 8,21% | - | - | 9,09% | - |  | - | - | - | 7,42% |
|  | GO:0006351 | transcription, DNA-dependent | 1,99% | - | 4,65% | 4,96% | - | - | - | - |  | - | - | - | - |
|  | GO:0006355 | regulation of transcription, DNA-dependent | 1,92% | - | 4,51% | 4,96% | - | - | - | - |  | - | - | - | - |
|  | GO:0010467 | gene expression | 8,12% | - | 11,19% | 11,64% | - | - | - | - |  | - | - | - | - |
|  | GO:0010468 | regulation of gene expression | 3,50% | 6,07% | 7,70% | 8,40% | - | - | 9,09% | - |  | - | - | - | 7,42% |
|  | GO:0016070 | RNA metabolic process | 3,51% | - | 5,96% | 6,11% | - | - | - | - |  | - | - | - | - |
|  | GO:0019222 | regulation of metabolic process | 3,62% | 6,36% | 7,85% | 8,59% | - | - | 9,09% | - |  | - | 6,83% | - | 7,86% |
|  | GO:0031323 | regulation of cellular metabolic process | 3,45% | 6,14% | 7,70% | 8,40% | - | - | 9,09% | - |  | - | - | - | 7,86% |
|  | GO:0032774 | RNA biosynthetic process | 1,99% | - | 4,65% | 4,96% | - | - | - | - |  | - | - | - | - |
|  | GO:0045449 | regulation of transcription | 3,29% | 5,77% | 7,56% | 8,21% | - | - | 9,09% | - |  | - | - | - | 7,42% |
|  | GO:0050789 | regulation of biological process | 4,17% | 6,94% | 8,58% | 9,16% | - | - | - | - |  | - | - | - | - |
|  | GO:0050794 | regulation of cellular process | 3,82% | 6,65% | 8,43% | 8,97% | - | - | 9,09% | - |  | - | - | - | 7,86% |
|  | GO:0065007 | biological regulation | 5,07% | 7,68% | 9,16% | 9,73% | - | - | 10,74% | - |  | - | - | - | - |
|  | GO:0006281 | DNA repair | 0,48% | - | - | - | - | - | - | 3,61% |  | - | - | - | - |
|  | GO:0002376 | immune system process | 0,32% | - | - | - | 2,73% | - | - | - |  | - | - | - | - |
|  | GO:0050896 | response to stimulus | 5,60% | - | - | - | 11,82% | - | - | 13,25% |  | - | - | - | - |
|  | GO:0006950 | response to stress | 2,71% | - | - | - | - | - | - | 8,43% |  | - | - | - | - |
|  | GO:0006974 | response to DNA damage stimulus | 0,48% | - | - | - | - | - | - | 3,61% |  | - | - | - | - |
|  | GO:0006855 | multidrug transport | 0,14% | - | - | - | - | - | - | 2,41% |  | - | - | - | - |
|  | GO:0009266 | response to temperature stimulus | 0,63% | 1,46% | - | - | - | - | - | - |  | 1,90% | - | - | - |
|  | GO:0009409 | response to cold | 0,38% | - | - | - | - | - | - | - |  | 1,14% | - | - | - |
|  | GO:0009628 | response to abiotic stimulus | 1,46% | - | - | - | - | - | - | - |  | 2,91% | - | - | - |
|  | GO:0009733 | response to auxin stimulus | 0,26% | - | - | - | - | - | - | - |  | - | - | 2,10% | - |
|  | GO:0009737 | response to abscisic acid stimulus | 0,27% | - | - | - | - | - | - | - |  | - | 1,71% | - | 1,75% |
|  | GO:0009738 | abscisic acid mediated signaling | 0,11% | - | - | - | - | - | - | - |  | - | 1,02% | - | 1,31% |
|  | GO:0065004 | protein-DNA complex assembly | 0,19% | - | - | - | - | 2,17% | - | - |  | - | - | - | - |
|  | GO:0046483 | heterocycle metabolic process | 0,75% | - | - | - | - | 3,62% | - | - |  | - | - | - | - |
| Molecular function | GO:0003676 | nucleic acid binding | 8,89% | 13,67% | 17,01% | - | - | 18,12% | - | 18,07% |  | - | - | - | - |
|  | GO:0003677 | DNA binding | 5,05% | 8,92% | 11,34% | - | - | - | 13,22% | - |  | - | - | - | 10,04% |
|  | GO:0003700 | transcription factor activity | 3,34% | 6,51% | 8,14% | - | - | - | 9,09% | - |  | - | 6,48% | - | 8,30% |
|  | GO:0008270 | zinc ion binding | 3,18% | 4,39% | - | - | - | - | - | - |  | - | - | - | - |
|  | GO:0043169 | cation binding | 4,89% | 6,65% | - | - | - | - | - | - |  | - | - | - | - |
|  | GO:0016667 | oxidoreductase activity, acting on sulfur group of donors | 0,13% | - | - | - | - | - | - | 2,41% |  | - | - | - | - |
|  | GO:0015297 | antiporter activity | 0,45% | - | - | - | - | - | - | 3,61% |  | - | - | - | - |
|  | GO:0051219 | phosphoprotein binding | 0,11% | - | - | - | - | - | - | - |  | 0,63% | 1,71% | - | 1,75% |
|  | GO:0004721 | phosphoprotein phosphatase activity | 0,93% | - | - | - | - | - | - | - |  | 2,02% | - | - | - |
|  | GO:0005544 | calcium-dependent phospholipid binding | 0,03% | - | - | - | - | - | - | - |  | - | - | 1,40% | - |
|  | GO:0015071 | protein serine/threonine phosphatase activity | 0,45% | - | - | - | - | - | - | - |  | - | - | - | 2,18% |
|  |  |  |  |  |  |  |  |  |  |  |  |  |  |  |  |

GO terms statistically enriched for specific SSRs subsets. Fisher’s exact test was performed between each SSRs subset versus the whole unigenes categorization ; only significant over-represented subset are reported (p<0,01). Analysis is displayed referring to “biological process” and “molecular function” classifications; “cellular component” is not reported due to the absence of particular enriched subsets.

**Electronic supplementary material**

- Additional file 1
- File format: .xls
- Title of data: EST assembly
- Additional file 2
- File format: .txt (fasta file)
- Title of data: 19055 unigenes
- Additional file 3
- File format: .ace (provided a .zip file)
- Title of data: ACE assembly file
  (EagleView available at http://bioinformatics.bc.edu/marthlab/EagleView)
- Additional file 4
- File format: .xls
- Title of data: primer pairs designed
- Additional file 5
- File format: .xls
- Title of data: full statistics on 300 SSR-containing loci
- Additional file 6
- File format: .xls
- Title of data: 19055 globe artichoke unigenes annotation

**References**

1.	Rottenberg A, Zohary D: **The wild ancestry of the cultivated artichoke**. *Genetic resources and crop evolution*: 1996, **43**(1):53-58.

2.	Lanteri S, Portis E: **Globe Artichoke and Cardoon**. In: *Vegetables I.* Edited by Springer, vol. 1. New York: Springer; 2008: 49-74.

3.	Kraft K: **Artichoke leaf extract - Recent findings reflecting effects on lipid metabolism, liver and gastrointestinal tracts.** *Phytomedicine* 1997, **4**(4):369-378.

4.	Gebhardt R: **Antioxidative and protective properties of extracts from leaves of the artichoke (*Cynara scolymus* L) against hydroperoxide-induced oxidative stress in cultured rat hepatocytes**. *Toxicology and applied pharmacology* 1997, **144**(2):279-286.

5.	Llorach R, Espin J, Tomas-Barberan F, Ferreres F: **Artichoke (*Cynara scolymus* L.) byproducts as a potential source of health-promoting antioxidant phenolics**. *Journal of agricultural and food chemistry* 2002, **50**(12):3458-3464.

6.	Wang M, Simon J, Aviles I, He K, Zheng Q, Tadmor Y: **Analysis of antioxidative phenolic compounds in artichoke (*Cynara scolymus* L.)**. *Journal of agricultural and food chemistry* 2003, **51**(3):601-608.

7.	McDougall B, King P, Wu B, Hostomsky Z, Reinecke M, Robinson W: **Dicaffeoylquinic and dicaffeoyltartaric acids are selective inhibitors of human immunodeficiency virus type 1 integrase**. *Antimicrobial agents and chemotherapy* 1998, **42**(1):140-146.

8.	Halvorsen B, Carlsen M, Phillips K, Bohn S, Holte K, Jacobs D, Blomhoff R: **Content of redox-active compounds (ie, antioxidants) in foods consumed in the United States**. *American journal of clinical nutrition* 2006, **84**(1):95-135.

9.	Lopez-Molina D, Navarro-Martinez M, Melgarejo F, Hiner A, Chazarra S, Rodriguez-Lopez J: **Molecular properties and prebiotic effect of inulin obtained from artichoke (*Cynara scolymus* L.)**. *Phytochemistry* 2005, **66**(12):1476-1484.

10.	Raccuia S, Melilli M: ***Cynara cardunculus* L., a potential source of inulin in the Mediterranean environment: screening of genetic variability**. *Australian journal of agricultural research* 2004, **55**(6):693-698.

11.	Lanteri S, Acquadro A, Comino C, Mauro R, Mauromicale G, Portis E: **A first linkage map of globe artichoke (*Cynara cardunculus* var. *scolymus* L.) based on AFLP, S-SAP, M-AFLP and microsatellite markers**. *Theoretical and applied genetics* 2006, **112**(8):1532-1542.

12.	Acquadro A, Lanteri S, Scaglione D, Arens P, Vosman B, Portis E: **Genetic mapping and annotation of genomic microsatellites isolated from globe artichoke**. *Theoretical and applied genetics*. Online first. DOI:10.1007/s00122-009-1005-6; 2009.

13.	Portis E, Mauromicale G, Mauro R, Acquadro A, Scaglione D, Lanteri S: **Construction of a reference molecular linkage map of globe artichoke (*Cynara* *cardunculus* var. *scolymus*)**. submitted.

14.	Tautz D, Renz M: **Simple sequences are ubiquitous repetitive components of eukaryotic genomes**. *Nucleic Acids Research* 1984(12):4127–4138.

15.	Tautz D: **Hypervariability of simple sequences as a general source for polymorphic DNA markers**. *Nucleic Acid Research* 1989(17):6443–6471.

16.	Powell W, Machray GC, Provan J: **Polymorphism revealed by simple sequence repeats**. *Trends in Plant Science* 1996, **1**(7):215-222.

17.	Toth G, Gaspari Z, Jurka J: **Microsatellites in different eukaryotic genomes: Survey and analysis**. *Genome research* 2000, **10**(7):967-981.

18.	Kumpatla SP, Mukhopadhyay S: **Mining and survey of simple sequence repeats in expressed sequence tags of dicotyledonous species**. *Genome* 2005, **48**(6):985-998.

19.	Morgante M, Hanafey M, Powell W: **Microsatellites are preferentially associated with nonrepetitive DNA in plant genomes**. *Nature genetics* 2002, **30**(2):194-200.

20.	Lanteri S, Saba E, Cadinu M, Mallica G, Baghino L, Portis E: **Amplified fragment length polymorphism for genetic diversity assessment in globe artichoke**. *Theoretical and applied genetics* 2004, **108**(8):1534-1544.

21.	Portis E, Barchi L, Acquadro A, Macua J, Lanteri S: **Genetic diversity assessment in cultivated cardoon by AFLP (amplified fragment length polymorphism) and microsatellite markers**. *Plant breeding* 2005, **124**(3):299-304.

22.	Lanteri S, Portis E: **Globe Artichoke and Cardoon**. In: *Vegetables I.* Edited by Springer, vol. 1. New York: Springer; 2008: 49-74.

23.	Acquadro A, Portis E, Albertini E, Lanteri S: **M-AFLP-based protocol for microsatellite loci isolation in *Cynara cardunculus* L. (Asteraceae)**. *Molecular ecology notes* 2005, **5**(2):272-274.

24.	Tang S, Knapp S: **Microsatellites uncover extraordinary diversity in native American land races and wild populations of cultivated sunflower**. *Theoretical and applied genetics* 2003, **106**(6):990-1003.

25.	Paniego N, Echaide M, Munoz M, Fernandez L, Torales S, Faccio P, Fuxan I, Carrera M, Zandomeni R, Suarez E *et al*: **Microsatellite isolation and characterization in sunflower (*Helianthus annuus* L.)**. *Genome* 2002, **45**(1):34-43.

26.	van de Wiel C, Arens P, Vosman B: **Microsatellite retrieval in lettuce (Lactuca sativa L.)**. *Genome* 1999, **42**(1):139-149.

27.	Portis E, Acquadro A, Comino C, Mauromicale G, Saba E, Lanteri S: **Genetic structure of island populations of wild cardoon [*Cynara cardunculus* L. var. *sylvestris* (Lamk) Fiori] detected by AFLPs and SSRs**. *Plant science* 2005, **169**(1):199-210.

28.	Metzgar D, Bytof J, Wills C: **Selection against frameshift mutations limits microsatellite expansion in coding DNA**. *Genome research* 2000, **10**(1):72-80.

29.	Zhang L, Zuo K, Zhang F, Cao Y, Wang J, Zhang Y, Sun X, Tang K: **Conservation of noncoding microsatellites in plants: implication for gene regulation**. *BMC Genomics* 2006, **7**:-.

30.	Li Y, Korol A, Fahima T, Nevo E: **Microsatellites within genes: Structure, function, and evolution**. *Molecular biology and evolution* 2004, **21**(6):991-1007.

31.	Richard G, Dujon B: **Association of transcripts from a group-I intron-containing gene with high sedimentation coefficient particles**. *Current genetics* 1997, **32**(3):175-181.

32.	Alba M, Santibanez-Koref M, Hancock J: **Amino acid reiterations in yeast are overrepresented in particular classes of proteins and show evidence of a slippage-like mutational process**. *Journal of molecular evolution* 1999, **49**(6):789-797.

33.	Shen H, Schmuck M, Pilz I, Gilkes N, Kilburn D, Miller R, Warren R: **Deletion of the linker connecting the catalytic and cellulose-binding domains of endoglucanase-a (cena) of cellulomonas-fimi alters its conformation and catalytic activity**. *Journal of biological chemistry* 1991, **266**(17):11335-11340.

34.	Pearson C, Edamura K, Cleary J: **Repeat instability: Mechanisms of dynamic mutations**. *Nature reviews genetics* 2005, **6**(10):729-742.

35.	Vancurova I, Vancura A, Lou W, Paine P: **Nucleoplasmins poly-glutamic acid tract is required for facilitated transport and enhances intranuclear binding**. *Molecular biology of the cell* 1995, **6**:1826-1826.

36.	Berger M, Sionov R, Levine A, Haupt Y: **A role for the polyproline domain of p53 in its regulation by Mdm2**. *Journal of biological chemistry* 2001, **276**(6):3785-3790.

37.	Gerber H, Seipel K, Georgiev O, Hofferer M, Hug M, Rusconi S, Schaffner W: **Transcriptional activation modulated by homopolymeric glutamine and proline stretches**. *Science* 1994, **263**(5148):808-811.

38.	Kolaczkowska A, Kolaczkowski M, Delahodde A, Goffeau A: **Functional dissection of Pdr1p, a regulator of multidrug resistance in *Saccharomyces cerevisiae***. *Molecular genetics and genomics* 2002, **267**(1):96-106.

39.	Perutz M, Johnson T, Suzuki M, Finch J: **Glutamine repeats as polar zippers - their possible role in inherited neurodegenerative diseases**. *Proceedings of the national academy of sciences of the united states of america* 1994, **91**(12):5355-5358.

40.	Leroy X.J. KLaMB: **Plant genomic instability detected by microsatellite primers.** In*.*, vol. 3. *Electronic Journal of Biotechnology* 2000.

41.	Guo H, Moose S: **Conserved noncoding sequences among cultivated cereal genomes identify candidate regulatory sequence elements and patterns of promoter evolution**. *Plant cell* 2003, **15**(5):1143-1158.

42.	Yang Y, Lai K, Tai P, Li W: **Rates of nucleotide substitution in angiosperm mitochondrial DNA sequences and dates of divergence between *Brassica* and other angiosperm lineages**. *Journal of molecular evolution* 1999, **48**(5):597-604.

43.	Mignone F, Grillo G, Licciulli F, Iacono M, Liuni S, Kersey P, Duarte J, Saccone C, Pesole G: **UTRdb and UTRsite: a collection of sequences and regulatory motifs of the untranslated regions of eukaryotic mRNAs**. *Nucleic acids research* 2005, **33**:D141-D146.

44.	Iglesias A, Kindlund E, Tammi M, Wadelius C: **Some microsatellites may act as novel polymorphic cis-regulatory elements through transcription factor binding**. *Gene* 2004, **341**:149-165.

45.	Martin P, Makepeace K, Hill S, Hood D, Moxon E: **Microsatellite instability regulates transcription factor binding and gene expression**. *Proceedings of the national academy of sciences of the united states of america* 2005, **102**(10):3800-3804.

46.	Temnykh S, DeClerck G, Lukashova A, Lipovich L, Cartinhour S, McCouch S: **Computational and experimental analysis of microsatellites in rice (*Oryza sativa* L.): Frequency, length variation, transposon associations, and genetic marker potential**. *Genome research* 2001, **11**(8):1441-1452.

47.	Lanteri S, Di Leo I, Ledda L, Mameli M, Portis E: **RAPD variation within and among populations of globe artichoke cultivar 'Spinoso sardo'**. *Plant breeding* 2001, **120**(3):243-246.

48.	Peakall R, Smouse P: **GENALEX 6: genetic analysis in Excel. Population genetic software for teaching and research**. *Molecular ecology notes* 2006, **6**(1):288-295.

49.	Smouse P, Peakall R: **Spatial autocorrelation analysis of individual multiallele and multilocus genetic structure**. *Heredity* 1999, **82**:561-573.

50.	Sneath PHA, Sokal RR: **Numerical taxonomy — the principles and practice of numerical classification.** In*.* W. H. Freeman: San Francisco; 1973.

51.	Rohlf FJ: **NTSYSpc Version 2.0: User Guide. Applied Biostatistics Inc.** In*.*; 1998.

52.	Altschul S, Madden T, Schaffer A, Zhang J, Zhang Z, Miller W, Lipman D: **Gapped BLAST and PSI-BLAST: a new generation of protein database search programs**. *Nucleic acids research* 1997, **25**(17):3389-3402.

53.	Min X, Butler G, Storms R, Tsang A: **OrfPredictor: predicting protein-coding regions in EST-derived sequences**. *Nucleic acids research* 2005, **33**:W677-W680.

54.	Beissbarth T, Speed T: **GOstat: find statistically overrepresented Gene Ontologies within a group of genes**. *Bioinformatics* 2004, **20**(9):1464-1465.
